# Detection of pharmaceuticals in wastewater effluents—a comparison of the performance of Chemcatcher® and polar organic compound integrative sampler

**DOI:** 10.1007/s11356-020-09077-5

**Published:** 2020-05-13

**Authors:** Anthony Gravell, Gary R. Fones, Richard Greenwood, Graham A. Mills

**Affiliations:** 1 Natural Resources Wales, Faraday Building, Swansea University, Singleton Campus, Swansea, SA2 8PP UK; 2 School of Earth and Environmental Sciences, University of Portsmouth, Burnaby Road, Portsmouth, PO1 3QL UK; 3 School of Biological Sciences, University of Portsmouth, King Henry Building, King Henry I Street, Portsmouth, PO1 2DY UK; 4 School of Pharmacy and Biomedical Sciences, University of Portsmouth, White Swan Road, Portsmouth, PO1 2DT UK

**Keywords:** Passive sampling, Chemcatcher^®^, POCIS, Pharmaceuticals, Screening, Wastewater effluent

## Abstract

Chemcatcher^®^ and POCIS passive sampling devices are widely used for monitoring polar organic pollutants in water. Chemcatcher^®^ uses a bound Horizon Atlantic™ HLB-L sorbent disk as receiving phase, whilst the POCIS uses the same material in the form of loose powder. Both devices (*n* = 3) were deployed for 21 days in the final effluent at three wastewater treatment plants in South Wales, UK. Following deployment, sampler extracts were analysed using liquid chromatography time-of-flight mass spectrometry. Compounds were identified using an in-house database of pharmaceuticals using a metabolomics workflow. Sixty-eight compounds were identified in all samplers. For the POCIS, substantial losses of sorbent (11–51%) were found during deployment and subsequent laboratory analysis, necessitating the use of a recovery factor. Percentage relative standard deviations varied (with 10 compounds exceeding 30% in both samplers) between individual compounds and between samplers deployed at the three sites. The relative performance of the two devices was evaluated using the mass of analyte sequestered, measured as an integrated peak area. The ratio of the uptake of the pharmaceuticals for the POCIS versus Chemcatcher^®^ was lower (1.84x) than would be expected on the basis of the ratio of active sampling areas (3.01x) of the two devices. The lower than predicted uptake may be attributable to the loose sorbent material moving inside the POCIS when deployed in the field in the vertical plane. In order to overcome this, it is recommended to deploy the POCIS horizontally inside the deployment cage.

**Electronic supplementary material:**

The online version of this article (10.1007/s11356-020-09077-5) contains supplementary material, which is available to authorized users.

## Introduction

Monitoring of various types of pollutants in water bodies is now of a global concern. The environmental concentration of several chemicals is regulated (i.e. have associated environmental quality standards) within various legislative directives and frameworks (e.g. European Union’s Water Framework Directive, (WFD)) (EC 2000). Many other substances are unregulated and are increasingly becoming of emerging environmental concern. For example, most pharmaceuticals, their metabolites and personal care products are unregulated (Archer et al. 2017; Petrie et al. 2015). Such classes of compounds are common in surface waters due to their continual discharge from wastewater treatment plants (WWTP) and other sources (e.g. aquaculture) (Archer et al. 2017; Petrie et al. 2015). There are over 5000 pharmaceutical products approved for use in Europe, yet typically, only a few hundred active compounds present in the products have been monitoring regularly in surface waters (Hughes et al. 2013). There is, therefore, a clear need for increased investigative monitoring activities to understand the occurrence and fate of these compounds and to ascertain the environmental risks that they may pose (Kosma et al. 2016; Petrie et al. 2013; Daughton 2016). In order to achieve this, advanced, high confidence, analytical workflows are needed to screen (e.g. targeted and untargeted analyses) for the presence or absence of a wide range of pollutants. Such analyses rely on the use of high-resolution accurate mass systems including, time-of-flight (TOF), quadrupole time-of-flight (Q-TOF) and Orbitrap instruments (Rimayi et al. 2019; Pinasseau et al. 2019; Abdallah et al. 2019). The use of high-resolution mass spectrometry is essential in order to obtain data on molecular ions, isotope patterns and fragment ions from the highly complex extracts, which contain numerous substances many of which will have similar molecular masses. When coupled to ultra-high performance liquid chromatography detection limits in the low ng L^−1^ range can be achieved using these systems (Acena et al. 2015).

In parallel to these sophisticated analytical protocols, effective environmental monitoring strategies are also needed to collect representative water samples. Most routine monitoring procedures rely on the infrequent (typically monthly) collection of low-volume (~ 1 L) spot (bottle or grab) samples of water. This approach has a number of shortcomings when the concentration of substances is known to fluctuate widely or there are stochastic inputs of pollutants over time (Castle et al. 2018a; Castle et al. 2019; Townsend et al. 2018). In order to overcome some of these difficulties, alternative monitoring strategies, such as the use of passive sampling devices has been proposed (Vrana et al. 2005). These devices are low-cost, non-mechanical and relatively simple in design; all have a receiving phase material with a high affinity for the chemical being monitored. Samplers can be deployed in the field for extended times (e.g. days to months) to continuously sequester pollutants. These devices can be used in various modes. In the qualitative mode, they can be used simply to screen for the presence or absence of pollutants (Rimayi et al. 2019). In the quantitative mode, they can yield time-weighted average (TWA) (Castle et al. 2018a; Castle et al. 2019; Townsend et al. 2018) or equilibrium concentrations (Vrana et al. 2005). To achieve this, the compound-specific sampler uptake rate (*R*_*s*_, mL day^−1^), diffusion coefficient or the sample/water partition coefficient (*K*_*sw*_) needs to be determined previously in either the laboratory or in situ in the field (Booij et al. 2007; Castle et al. 2018b; Petrie et al. 2016). A wide range of devices now exists capable of sequestering most classes of environmental pollutants (Vrana et al. 2005). For non-polar pollutants, semi-permeable membranes devices (SPMDs) or more recently polymeric sheets (e.g. low-density polyethylene or silicone rubber) are used with or without added performance reference compounds (PRCs) (Lohmann et al. 2012; Taylor et al. 2019). For semi-polar and polar moieties, three designs of the sampler are typically used; the polar organic compound integrative sampler (POCIS) (Godlewska et al. 2019), the polar version of the Chemcatcher^®^ (Petrie et al. 2016), and more recently the organic version (o-DGT) of the diffusion gradients in thin film device (Challis et al. 2016).

The POCIS was designed to sequester polar analytes (~log *K*_*ow*_ 0.1–3.0), although semi-polar compounds with higher values (e.g. hormones and steroids) have also been shown to accumulate (Alvarez et al. 2013; Creusot et al. 2014). It comprises a loose receiving phase sorbent (typically 200 mg in the standard device) enclosed between two diffusion membranes (usually microporous polyethersulphone (PES), 0.1 μm pore size). To prevent loss of sorbent in use, two ‘O’ rings (typically stainless steel) are used to firmly sandwich the PES membranes. These rings are fixed in place using nuts and bolts (Alvarez et al. 2004). Larger substances (cross-sectional diameters > 0.1 μm) such as bacteria, macromolecules and particulate material, are therefore, excluded from sequestration by the device (Petty et al. 2004). Due to the low-energy surface properties of the PES, biofouling on the surface of the membrane is also minimised during extended field deployments. The standard device has an effective sampling surface area of 45.8 cm^2^ (Godlewska et al. 2019). Two designs of POCIS are frequently used, each having a different receiving phase sorbent. A configuration for hormones, pesticides and chemicals found in wastewater comprises a tri-phasic mixture of Isolute ENV^+^ polystyrene divinylbenzene resin (80% by weight) and Ambersorb 1500 carbon, lightly dispersed on S-X3 Biobeads (20% by weight) (Godlewska et al. 2019). The ‘pharmaceutical POCIS’ contains only a hydrophilic–lipophilic balanced sorbent (Waters Oasis^®^ HLB). This water-wettable–reversed-phase sorbent comprises a macroporous poly (divinylbenzene-co-*N*-vinylpyrolidone) polymer. It exhibits both hydrophilic and lipophilic retention characteristics and is capable of adsorbing both polar and semi-polar compounds (Godlewska et al. 2019). Oasis^®^ HLB is the most frequently used sorbent and has been used for monitoring industrial chemicals, pesticides, pharmaceuticals and personal care products (García-Córcoles et al. 2019). Typical sampler deployment periods are 14–21 days, but longer periods (~ 30 days) have been reported (Morin et al. 2012).

The polar Chemcatcher^®^ is similar in construction, comprising a three-component PTFE body, and a bound receiving phase that is overlaid with a thin PES diffusion membrane (0.2 μm pore size, 47 mm diameter) (Vrana et al. 2007). The current commercially available version has an active sampling area of 15.2 cm^2^. Several types of immobilised receiving phase have been used, including 3 M Empore™ disks (e.g. a 47-mm modified styrene-divinylbenzene (SDB-RPS or SDB-XC) or anion-exchange sorbent disk (Charriau et al. 2016; Lissalde et al. 2016; Townsend et al. 2018) and more recently the Horizon™ Technology Atlantic disk (e.g. a 47 mm Oasis^®^ HLB-L disk) (Petrie et al. 2016). For most applications, deployment periods ranged from 7 to 30 days, with 14 days being the most common (Castle et al. 2018b; Lissalde et al. 2016; Townsend et al. 2018). The accumulation of specific compounds into the device is altered by the choice of receiving phase sorbent used.

Recently, an organic version of the well-established DGT sampler has been developed (Challis et al. 2016). The o-DGT is made from several layers where compound uptake rates are different. During the deployment, analytes diffuse through the diffusive boundary layer and a material diffusion layer comprising a filter membrane and hydrogel; lastly, a specific binding layer sequesters the compound. o-DGT has been used to measure various organic compounds such as bisphenols, pesticides and phenols, using various binding material such as activated charcoal, Oasis^®^ HLB, Oasis^®^ HLB-MAX, and a molecularly imprinted polymer (Challis et al. 2016; Dong et al. 2014; Guibal et al. 2017; Zheng et al. 2015). The sampler has a normalised surface area sampling rate of between 0.54–5.74 mL day^−1^ cm^−2^ that is comparable with the other designs of polar passive samplers. A benefit of the o-DGT over other passive samplers is that a simpler calibration is needed. In this case, the diffusional characteristics of individual compounds are obtained in the laboratory as a fundamental physical property (Challis et al. 2016).

Although many field studies have been undertaken with these three designs of polar devices, there has been very little work directly comparing their functionalities. Challis et al. (2018) investigated the field performance (for the uptake of pharmaceuticals and polar pesticides in an agricultural river catchment in Manitoba, Canada) of the POCIS alongside the o-DGT together with the collection of spot samples of water. Results showed that there was an underestimation (2.3-fold) of concentrations with the POCIS compared with the o-DGT. This was likely due to the water flow and boundary layer effects associated with the use of the POCIS. Their work, however, showed the o-DGT to be a robust, sensitive and reliable monitoring tool for these classes of analytes. Recently, Buzier et al. (2019) in a laboratory study looked at the effects of water flow (quiescent and flow 2–18 cm s^−1^) on the uptake of ten pharmaceuticals by both the POCIS (with added PRCs) and o-DGT. Results showed that both devices gave similar results under flow conditions, but the o-DGT is more efficient under quiescent conditions. This work showed, however, that the o-DGT suffered from poor sensitivity due to the size of the active sampling area (3.1 cm^2^), but that this could potentially be overcome in the future by the use of a larger sampler (Urik and Vrana 2019).

We undertook a similar study, but here comparing the field performance and sampling efficiency of the Chemcatcher^®^ versus the POCIS. Both devices contained the same type (Oasis^®^ HLB) and amount (200 mg) of receiving phase sorbent but were retained in different formats (bound and unbound). Hence, it was thought that both polar devices would behave similarly. Samplers were exposed for 21 days in the effluent of three different WWTP in Wales, UK. We used liquid chromatography/quadrupole-time-of-flight-mass spectrometry (LC/Q-ToF-MS) analysis combined with various statistical tools, to investigate the reproducibility of the integrated chromatographic peak areas of targeted pharmaceutical substances identified during the screening of extracts against an in-house database of pharmaceutical compounds. It was not the objective of this study to measure the compound specific uptake rate (*R*_*s*_) for each of the pharmaceutical compounds identified, as this was beyond the scope of the work and would also be prohibitive based on cost and labour considerations. Hence, TWA concentrations were not estimated in this study.

## Materials and methods

### Glassware, reagents and standards

Reagents and solvents were of analytical reagent grade or better. Acetone, ammonium acetate, ammonium formate, dichloromethane, formic acid, methanol, methyl-tert-butyl ether (MTBE), Sylon CT™ solution (5% dimethylchlorosilane in toluene) and toluene were obtained from Fisher Scientific, UK (Loughborough, Leicestershire, UK) or Sigma-Aldrich (Gillingham, Dorset, UK). Pharmaceutical standards (164 compounds, 5 mg, purity ≥ 95%) used to develop the LC/Q-ToF-MS database were purchased from Sigma-Aldrich (Table S1). Stock and dilute working solutions were prepared as described in the Supplementary Material and kept at 3–5 °C for up to 1 month. Ultrapure water (ELGA Purelab Ultra, Marlow, Buckinghamshire, UK) was used in all laboratory procedures. All glassware was silanised (5% dimethylchlorosilane in toluene). This was to reduce surface activity of the glass surfaces and thereby prevent any loss of analytes through adsorption.

### Passive samplers

#### POCIS

Supor^®^ 100 PES membrane (30 cm × 15 m, 0.1 μm pore size) was purchased from Pall Corporation (Portsmouth, UK). Oasis^®^ HLB sorbent (receiving phase) was purchased from Waters Chromatography Ireland Ltd. (Dublin, Ireland) as a loose powder. Stainless steel (316 grade) ‘O’ rings, were made by A.T. Engineering (Tadley, Hampshire, UK). PES membranes (18 cm × 9 cm) were cut from the roll and cleaned prior to use to remove polyethylene glycol impurities present in their manufacture. The procedure used was similar to that described by Guibal et al. (2015). The cut PES membranes were soaked twice (24 h, 40 °C) in methanol/water (20%) and then this procedure was repeated using only a methanol wash. Cleaned membranes were placed on aluminium foil and dried (~ 8 h). Once dry, they were placed in a storage can (stainless steel), being flushed with argon and kept at − 18 °C until use. Six grams of Oasis^®^ HLB sorbent (enough for 30 devices) was placed in a glass chromatography column. The sorbent was washed (250 mL) in sequence with methanol, methyl-tertiary-butyl ether (MTBE), dichloromethane and again methanol. The cleaned sorbent was removed, thoroughly dried in a centrifugal rotary vacuum evaporator (Genevac Rocket™, Genevac Ltd., Ipswich, UK) and then placed in a sealed glass jar until use. Two hundred (± 2.0) milligram of the purified Oasis^®^ HLB was used for each POCIS device. The sorbent was spread out and sandwiched between two PES membranes and clamped together using two ‘O’ rings. The ‘O’ rings were secured firmly using three self-locking stainless steel bolts (Fig. S1). Once prepared devices were wrapped in aluminium foil and stored in clean, sealable metal cans at − 20 °C until deployment. PRCs were not used in this trial as their effectiveness with polar passive samplers is not proven (Buzier et al. 2019).

#### Chemcatcher^®^

Three-component PTFE Chemcatcher^®^ bodies (Atlantic design, A.T. Engineering) (Fig. S2) were soaked overnight in 10% Decon^®^ detergent, washed thoroughly in water then rinsed in methanol. The components were then allowed to dry. Horizon Atlantic™ HLB-L disks (47 mm containing 200 mg of sorbent) (ARC Sciences Ltd., Alton, UK) were used as the receiving phase. Disks were soaked overnight in methanol and allowed to dry. The disks were then placed in a vacuum filter funnel manifold and methanol (50 mL) followed by water (50 mL) allowed to pass through under gravity. The conditioned disks were removed and placed in water until use. Disks (51 mm diameter) of Supor^®^ 200 polyethersulphone membrane (0.2 μm pore size) (Pall Corporation) were punched from a roll and washed and stored as described for the preparation of the POCIS membranes. The prepared HLB-L disks were placed on the support body of the Chemcatcher^®^ and a clean PES membrane placed on the top of the disk. The PTFE retaining ring, that holds the disk and membrane in place, was screwed onto the Chemcatcher^®^ body and firmly tightened. It was important to ensure that no air was trapped between the two layers. The assembled Chemcatcher^®^ samplers were stored (3–5 °C) submerged in water until use. PRCs were not used in the trial. Prior to transport to the field, a clean PTFE lid was fitted to each device.

### Extraction

#### POCIS

After deployment, POCIS was removed from its deployment holder, cleaned with water to remove any external fouling and allowed to dry overnight. The device was opened, and the exposed HLB sorbent was dried overnight. The sorbent was transferred from the PES membrane by carefully brushing into a pre-weighed glass vial (15 mL). The sorbent is electrostatic when dried, so special precautions were undertaken to minimise losses during the transfer operation. The mass of sorbent recovered from each deployed POCIS was recorded. The sorbent was then quantitatively transferred to a polypropylene solid-phase extraction (SPE) cartridge (15 mL) fitted with a PTFE frit. Using an SPE vacuum elution system, the sequestered compounds were eluted using methanol (40 mL) at a flow rate of ~ 1 mL min^−1^ with the eluate collected into a glass vial (50 mL). The methanol was reduced in volume to ~ 0.5 mL using a Genevac Rocket™ set to the low boiling point mode. Each extract was resolvated with methanol to 1.0 mL, and then transferred to a silanised glass vial (2 mL). Prior to analysis, the extract was diluted (× 10) using mobile phase B (an aqueous solution of 10% of methanol, 5 mM ammonium formate and 0.01% formic acid). The dilution step minimised matrix effects during the subsequent instrumental analysis.

#### Chemcatcher^®^

After exposure, Chemcatcher^®^ samplers were carefully cleaned with water to remove any bio-fouling and disassembled. The PES membrane was discarded. Residual moisture was removed from the HLB-L disk by drying (~ 1 h) on a vacuum manifold. Analytes were then extracted under gravity using small aliquots of methanol (total 40 mL), being collected into glass vials (50 mL). The subsequent analytical steps were as described above for the POCIS.

### Field trial sites and sampler deployments

Chemcatcher^®^ and POCIS devices were deployed in the final effluent channel (in order to minimise ragging and biofouling of the samplers) at three similarly designed WWTP located in South Wales, UK, covering the areas of Carmarthen, Gowerton (west Swansea) and Llanelli. Permission was obtained from the site operators to deploy the samplers. WWTP A (latitude 51.8358 longitude − 4.3268) treats wastewater from a population equivalent of ~ 22,000 and effluent from a local General Hospital, located 4.5 km north of the works. WWTP B (latitude 51.6545 longitude − 4.0323) treats wastewater from a population equivalent of ~ 50,000. WWTP C (latitude 51.6636 longitude − 4.1098) treats wastewater from a population equivalent of ~ 55,000 and also effluent from a local hospital. At WWTP B two deployment rigs (WWTP B (1) and WWTP B (2)) that were placed adjacent to each other were used in order to assess the variability of uptake between samplers at the same site. Triplicate Chemcatcher^®^ (Fig. S3) and POCIS samplers (Fig. S4) were used at each site. The Chemcatcher^®^ transport lid was removed and the POCIS was taken from its storage can and attached to stainless steel holders then placed inside a stainless steel cage for protection. Chain (5 mm) was used to fix the deployment cages to a platform covering the final effluent channels at the three sites (Fig. S5), where the average flow rates were 81 L s^−1^ (WWTP A), 194 L s^−1^ (WWTP B) and 255 L s^−1^ (WWTP C). All samplers were deployed for 21 days between 4th and 25th of August 2014. This time period was selected to ensure adequate sequestration of analytes for subsequent analysis. Over this period, it was expected that most compounds would still be in the time integrative regime whilst a few may have approached equilibrium. At each site, during deployment and retrieval operations, a field blank sampler for each design was exposed to the air then resealed.

### LC/Q-ToF-MS analysis

Chromatographic separation of the substances in the pharmaceutical standards mix (Table S1) and those found in the extracts from the passive sampling devices (deployed and field blanks) was carried out using an Agilent 1290 Infinity ultra-high performance liquid chromatography (UHPLC) (Agilent Technologies, Santa Clara, USA) system fitted with an Atlantis T3 (2.1 mm i.d. × 150 mm, 3.5 μm particle size) (Waters, Elstree, UK) column. The UHPLC system was interfaced to an Agilent G6540A Q-ToF-MS equipped with a dual spray jet stream electrospray ionisation source (ESI). A reference mass solution was injected continuously (10 μL min^−1^) to the reference sprayer (using nitrogen gas at 5 psi). The second sprayer delivered the column eluent to the ESI source. The Q-ToF MS instrument gathered data (2 GHz) using the extended dynamic range mode with both positive and negative ion ESI for the targeted chemicals. Sample data (molecular adduct ions and their isotopes) was acquired (Agilent Mass Hunter acquisition software (rev. B.06.01)) using ‘all Ions MS/MS’ mode (i.e. alternating low/high collision cell energy setting). All stored data was then adjusted using the reference lock mass correction. Further details of the UHPLC and Q-ToF-MS analytical conditions are given in Tables S2 and S3.

Data analysis to identify and quantify the pharmaceutical compounds in the Chemcatcher^®^ and POCIS extracts followed a typical LC/MS-based metabolomics workflow. This involved (i) compound extraction from targeted analysis (ii) alignment of peak retention time (iii) integration of peak area and (iv) export of data for statistical evaluation using Agilent Mass Profinder in conjunction with Microsoft Excel and Minitab software (Gravell 2017). Further details of the development of the compound database library and procedures used are given in the Supplementary Material and Table S4.

## Results and discussion

As POCIS and Chemcatcher^®^ are of similar design and use the same receiving phase sorbent, it was expected that both would sequester a similar range of chemicals, but with varying amounts, at the three WWTP deployment sites. There was a difference in the pore size of the PES membrane; the POCIS used 0.1 μm pore size, whilst the Chemcatcher^®^ used 0.2 μm pore size, although this difference was not thought to significantly influence uptake rates due to the small molecular size (< 1000 Da) of the compounds sequestered. Both types of samplers were deployed in the same protective cage; hence, any effects on uptake due to the design of the deployment apparatus were minimised. No sampler was lost during the field trial. There was limited fouling of the PES membrane even after a 3-week deployment in the final effluent channel. No pharmaceutical substances that were in the compound database library were detected in the exposed field blank samplers.

### Sorbent receiving phase

Upon disassembly of the POCIS, it was noted that the HLB sorbent was unevenly distributed between the PES membranes (Fig. S6). It was evident that the loose powder had flowed and sagged toward the base of the device during vertical deployment. This potentially reduced the active sampling surface area and could have led to increased variability in uptake rate for the pharmaceutical compounds (Mills et al. 2014). Such effects could be reduced by placing the samplers in the horizontal plane during field deployments (Seen et al. 2014). In the Chemcatcher^®^ the HLB sorbent in the receiving phase disk is immobilised and hence the active sampling area should have remained constant during the field deployments (Fig. S7).

We found losses of sorbent occurred when disassembling the POCIS in the laboratory and/or during the deployment (Gravell 2017). The losses varied considerably between devices and across all three deployment sites (Table 1). An overall average of 70% (RSD = 15%) of sorbent was recovered from the original 200 mg that was added to each device. Significantly lower amounts of sorbent, 104 mg and 97 mg, were recovered from the POCIS deployed at WWTP A (sampler 1) and WWTP C (sampler 2) respectively. The reasons for these high losses is unknown. To our knowledge, such losses have not been reported by other workers who have used the POCIS in field studies; it is generally assumed the same mass of sorbent initially added during the preparation stage is recovered during disassembly and analysis. Furthermore, the HLB sorbent when dry becomes electrostatic and special precautions may be required to prevent static build-up; this effect is often neglected or not mentioned in the published literature. We carefully removed the HLB sorbent from the PES membrane prior to extraction and analysis. Often, however, it is suggested that the PES membrane from POCIS deployments be placed directly in a glass funnel and methanol used to wash the sorbent off into a receptacle. This can result in the desorption of compounds which have been adsorbed onto the PES membrane during deployment (Vermeirssen et al. 2012). POCIS may be considered a mono-phasic sampler (Fauvelle et al. 2014). Hence, equations used to calculate uptake rates consider only what is adsorbed onto the sorbent. Inaccurate results may be obtained if compounds that are adsorbed onto the membrane are also measured and included in any uptake rate estimations. This contrasts the Chemcatcher^®^ as this considers only compounds that have passed through the membrane and subsequently adsorbed onto the sorbent and glass fibre matrix of the disk (Gravell 2017).

**Table 1:** Amount and percentage recovery of HLB sorbent recovered from POCIS deployed at the three WWTP sites in South Wales. Amount of sorbent originally added was 200 mg (A)

Site	Amount of sorbent recovered (mg) (B)	Percentage of sorbent recovered	Recovery correction factor A/B
WWTP A, sampler 1	104	52	1.92
WWTP A, sampler 2	141	71	1.42
WWTP A, sampler 3	146	73	1.37
WWTP B1, sampler 1	144	72	1.39
WWTP B1, sampler 2	146	73	1.37
WWTP B1, sampler 3	126	63	1.59
WWTP B2, sampler 1	147	74	1.36
WWTP B2, sampler 2	151	78	1.32
WWTP B2, sampler 3	143	71	1.40
WWTP C, sampler 1	178	89	1.12
WWTP C, sampler 2	97	49	2.06
WWTP C, sampler 3	147	73	1.36

As there were substantial losses of sorbent from the POCIS during the deployment and analysis steps a recovery correction factor was used (Table 1). This was applied to all the compound integrated peak areas derived from the LC/Q-ToF-MS analyses. The use of this correction factor permitted the direct comparison of the compound uptake in both types of samplers. It was assumed that no losses of sorbent occurred with the Chemcatcher^®^ as the material is immobilised within a glass fibre matrix. The use of commercially available and quality-controlled receiving phases as used with the Chemcatcher^®^ allows for simplicity of use when compared with some other designs of passive sampling devices.

### Identification of pharmaceutical compounds

Using the compound database library, 72 pharmaceuticals were identified in the passive sampler extracts (Table 2). Sixty-eight compounds were present at all three sites and in both types of samplers. Atorvastatin, naproxen and terbinafine were found only at the WWTP A site, whilst piroxicam was found only at the WWTP C site. Sixty-seven compounds were identified in the positive ion mode, 5 compounds in the negative ion mode with 13 substances detectable using both ionisation modes (Table 2). For both samplers, the peak areas obtained from positive ion mode for these 13 compounds were generally higher than that obtained from negative ion mode; therefore, the positive ion data were selected for subsequent statistical analysis for comparing the performance of the two devices. Of the five chemicals identified (negative ion mode only), four were acidic and hence did not efficiently ionise in positive ion mode due to their very low proton affinities. The pharmaceutical compounds found at the wastewater sites covered a wide variety of therapeutic drug classes. Many of these substances have been detected previously using the POCIS with an HLB receiving phase (Baz-Lomba et al. 2017; Godlewska et al. 2019; Guibal et al. 2018; Harman et al. 2012; Morin et al. 2013). Similarly, these compounds have also been detected in wastewater effluent and receiving surface waters using the polar Chemcatcher^®^ (Petrie et al. 2016; Rimayi et al. 2019).

**Table 2:** Percentage relative standard deviations (*n* = 3) based on the integrated peak areas obtained for 72 pharmaceuticals identified using the compound database library in Chemcatcher^®^ and POCIS sampler extracts at the four different deployments using LC/Q-ToF-MS. POCIS data corrected for the loss of sorbent during deployment and analytical transfer operations. The table was adapted from Gravell (2017)

Compound	WWTP A Chemcatcher^®^	WWTP A POCIS	WWTP B (1) Chemcatcher^®^	WWTP B (1) POCIS	WWTP B (2) Chemcatcher^®^	WWTP B (2) POCIS	WWTP C Chemcatcher^®^	WWTP C POCIS
Alfuzosin	27.9	12.5	1.8	6.4	16.2	5.7	17.4	1.5
Alverine	12.9	7.4	11.6	5.1	5.6	3.8	27.2	8.1
Amisulprideb	12.8	5.7	9.1	5.8	6.5	2.6	3.9	3.4
Amitriptyline	15.8	18.7	9.0	3.5	6.2	4.9	4.8	14.2
Atenolol	7.5	7.2	8.4	3.4	4.6	1.9	3.5	3.1
Atorvastatin	21.8	32.4	nd	nd	nd	nd	nd	nd
Betamethasone 17 valerate	23.1	32.7	14.6	19.2	15.2	31.5	12.4	8.1
Bezafibrate	18.3	6.9	8.0	6.6	6.9	3.1	3.2	10.1
Bisoprolol	13.9	4.2	9.3	3.7	10.2	3.3	3.6	4.0
Carbamazepine	13.3	6.9	8.9	4.1	7.3	1.8	3.0	3.0
Cefalexin	14.2	4.9	9.9	4.8	11.0	6.1	3.5	9.4
Celiprololb	16.9	6.8	7.6	4.6	7.9	1.5	1.3	2.1
Cetirizine	13.0	4.0	6.2	3.1	7.8	2.5	3.0	4.5
Chlorpheniramine	19.2	8.6	25.3	15.1	5.9	33.2	32.1	20.9
Citalopram	14.1	8.2	7.8	3.8	6.8	4.2	3.8	11.8
Clarithromycinb	37.5	5.8	5.2	2.7	4.9	4.7	4.9	15.7
Clopidogrel	12.3	19.2	6.3	3.3	7.2	1.5	9.5	9.5
Cyclizine	13.2	4.5	6.8	1.7	9.1	2.3	3.6	5.8
Diclofenac	11.0	5.9	7.9	4.6	3.8	3.3	2.9	2.0
Diltiazem (cis)	24.6	7.4	7.1	3.0	8.8	4.2	4.0	12.4
Dipyridamole	24.0	5.0	9.1	6.5	6.2	5.4	5.0	3.7
Doxazosin	49.5	30.2	28.3	14.3	24.0	20.6	2.7	8.3
Erythromycinb	27.1	5.4	6.9	3.3	8.3	4.1	0.8	13.5
Fexofenadineb	15.1	5.0	8.2	3.8	8.1	3.4	3.4	7.0
Flecainideb	25.7	3.9	6.8	4.9	22.5	3.2	5.4	8.2
Fluconazoleb	8.5	10.7	5.0	7.4	7.4	3.1	9.0	3.5
Furosemide	3.8	15.6	0.6	6.6	7.9	9.4	6.2	12.5
Gliclazide	12.9	9.9	24.5	3.4	6.1	4.5	2.5	2.1
Ibuprofena	6.2	10.4	4.8	7.5	5.0	7.8	6.2	11.7
Indapamidea	4.9	9.0	3.2	7.1	3.1	4.5	2.7	1.3
Irbesartanb	14.1	3.2	7.0	3.5	7.7	2.7	4.1	4.9
Ketoconazole	26.3	5.8	11.4	3.3	8.3	2.7	3.5	14.2
Ketoprofen	12.4	17.6	6.7	2.4	24.2	14.9	24.6	7.3
Labetalol	12.0	1.1	10.1	3.7	7.1	1.2	0.5	11.0
Lamotrigine	9.5	9.6	10.5	3.6	5.5	1.1	2.0	2.0
Lansoprazole	10.1	8.2	10.4	3.9	8.8	2.2	3.1	9.8
Lidocaine	12.3	7.0	6.8	1.8	8.6	1.8	3.8	3.2
Loperamide	18.3	6.7	14.2	2.5	3.2	5.7	11.7	13.7
Loratadine	23.0	97.7	22.0	164.7	18.2	5.1	15.0	22.1
Losartan	14.4	4.0	10.8	5.4	1.0	6.3	7.0	3.8
Mefenamic acida	11.0	2.9	8.6	5.7	7.3	3.2	2.4	5.1
Metoclopramide	12.8	8.4	10.3	2.1	4.6	1.8	2.7	3.7
Metoprolol	13.4	10.6	7.5	5.5	9.5	1.2	3.5	3.7
Mirtazapine	11.7	3.6	10.0	4.4	8.5	3.0	3.1	3.6
Naproxena	10.7	34.6	nd	nd	nd	nd	nd	nd
Nifedipine	32.0	45.7	47.8	18.2	24.0	13.4	9.3	28.5
Omeprazole	16.4	1.8	6.9	3.9	7.4	6.6	6.0	10.3
Oxprenololb	2.5	7.9	6.7	7.1	4.8	4.0	3.1	4.4
Pantoprazole	11.7	12.8	26.6	6.3	20.3	5.1	7.1	8.8
Phenytoin	13.1	13.9	6.5	6.6	8.6	1.2	4.6	4.4
Piroxicam	nd	nd	nd	nd	nd	nd	3.7	3.2
Procyclidine	20.2	8.1	6.0	3.9	7.8	6.5	6.5	7.9
Propranolol	13.6	3.3	7.7	3.8	6.9	2.4	4.4	6.5
Quinine	15.6	4.7	11.3	3.4	14.6	3.7	3.3	5.5
Ranitidine	20.7	32.2	31.2	19.2	16.5	15.8	1.9	21.8
Salbutamol	4.2	10.7	9.2	1.4	43.1	4.6	4.7	5.8
Salicylic acida	8.7	15.1	13.3	2.9	12.6	11.4	3.9	14.1
Sertraline	20.6	22.1	11.8	3.4	1.7	4.1	6.2	11.2
Sotalolb	9.2	8.5	7.9	4.0	5.7	2.5	3.1	2.5
Sulfasalazine	10.9	4.7	6.7	5.7	5.3	0.9	3.8	5.6
Sumatriptanb	11.6	9.0	14.4	5.3	5.2	3.9	2.5	5.9
Tamsulosin	28.4	5.0	29.1	5.7	11.1	15.9	23.5	27.6
Telmisartan	14.9	11.1	7.6	5.2	4.9	1.2	6.0	8.9
Terbinafine	35.8	17.0	nd	nd	nd	nd	nd	nd
Timolol	32.2	19.5	18.5	12.3	22.2	15.8	8.3	10.2
Tramadolb	12.8	5.9	8.2	4.4	8.0	1.5	3.5	0.2
Trazodone	27.5	25.0	6.6	3.8	10.3	0.6	6.3	7.0
Trimethoprim	13.4	8.4	7.5	3.6	8.1	0.9	3.8	0.2
Valsartanb	23.6	4.5	18.1	17.2	6.2	8.4	5.0	13.1
Venlafaxine	13.5	6.8	7.8	4.3	7.4	2.5	3.4	0.3
Verapamil	31.0	12.2	2.2	2.7	7.4	5.1	3.0	12.7
Warfarin	16.8	9.0	10.1	5.6	6.6	2.5	14.8	8.7

^a^Obtained from negative ion data

^b^Identified in both positive and negative ionisations

RSDs expressed as a percentage. All the other compounds were identified in positive ionisation. nd, not detected in extract

The integrated peak areas (corrected for the loss of HLB sorbent in the POCIS) obtained for each pharmaceutical compound detected were averaged (*n* = 3), and percentage relative standard deviations (% RSD) were calculated (Table 2). Larger peak areas were obtained for the POCIS due to its larger active sampling area (3.01 times bigger). Percentage RSD’s varied between individual compounds and between samplers deployed at the three sites, including the duplicate cages at WWTP site B. RSD’s (%) obtained from the positive ion analysis were generally higher for the Chemcatcher^®^ sampler at two sites (WWTP A and B), but substantially lower than POCIS for the WWTP C site. The variability in recovery of the sorbent obtained from the POCIS for site C may have led to the higher % RSD’s. There were 32 compounds where the % RSD exceeded 15% for the POCIS extracts. This was compared with 56 compounds for the Chemcatcher^®^. Lower RSD’s would have been expected for the Chemcatcher^®^ sampler as it uses a bound-receiving phase sorbent and thereby should have increased the reproducibility of the device.

One possible reason contributing to this observation was the smaller peak areas, typically 2–3 times smaller than the POCIS. Only 10 compounds exceeded 30% RSD in both samplers with the largest error observed for loratadine (165% RSD) in the POCIS. For the Chemcatcher^®^ sampler, RSD’s obtained from negative ion analysis were generally lower across all sites. One compound (naproxen) in the POCIS, exceeded 30% RSD in negative ion mode. As the mass spectrometric response for each pharmaceutical compound differs significantly this will also influence the % RSD obtained, as smaller responses will generally lead to larger overall errors.

### Comparison of uptake of pharmaceuticals by Chemcatcher^®^ versus POCIS

The integrated peak areas for each of the 68 compounds identified at all sites were used to compare the performance of the two devices. As both data sets had an independent error distribution a simple linear regression analysis was not appropriate; instead an orthogonal regression or Deming regression (Cornbleet and Gochman 1979) was used. This approach is different than with ordinary least squares regression. With least square regression, the (*x*) or predictor value is a fixed variable with no error. The (*y*) dependent variable has all of the error is associated with it. The fitted line with the least square regression always gives the minimum deviation of the *y* values from the fitted line. The fitted line in orthogonal regression corresponds to the minimum deviations at right angles to the fitted line. This is equivalent to the first principal component (Cornbleet and Gochman 1979). With orthogonal regression, the interpretation of the regression parameters is similar to that for the least squares. In order to fit an orthogonal regression, it is necessary to have an estimate of the variance ratio based on the experimental error for the two samplers. This was obtained using a data set (*n* = 8796 observations) based on the integrated peak areas of the unknown compounds also found in the LC/Q-ToF-MS analyses at the same sites as for the targeted pharmaceutical analyses. The estimates of the experimental errors were calculated using a two-way analysis of variance of the data (Table S5). The variance associated with the compound, site and a compound-site interaction term was removed from the total variation and the residual error was representative of the experimental error. The variance ratios (POCIS:Chemcatcher^®^) for the untransformed and log_10_ transformed data were 14.59 and 1.92 respectively.

**Fig. 1:**
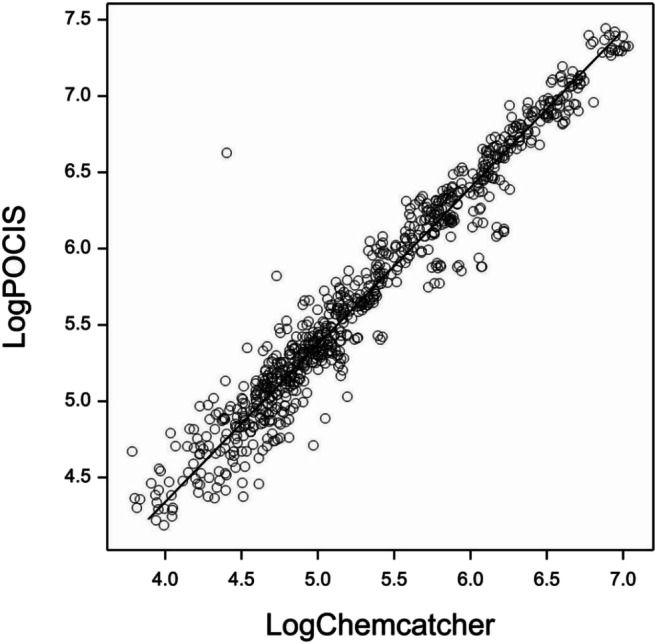
Fitted line (―) from the log_10_ transformed orthogonal regression analysis for the integrated peak areas (○) for the 68 pharmaceutical compounds found at all sites (including duplicate deployment at WWTP 2 site) using the Chemcatcher^®^ and POCIS. The regression equation was log_10_POCIS = 0.264 + 1.022 log_10_Chemcatcher

Normal probability plots for the untransformed data showed a large skew (Fig. S8) with log_10_ transformation rendering the data approximately normal (Fig. S9). Therefore, subsequent orthogonal regression analysis used the log_10_ transformed data. The regression equation was log_10_POCIS = 0.264 + 1.022 log_10_Chemcatcher and the fitted line is shown in Fig. 1. Taking the anti-log_10_ of the intercept gave the ratio of the uptakes by POCIS and Chemcatcher^®^ of 1.84x. The analysis of variance output for the transformed regression is shown in Table S6.

The ratio of the uptake of the pharmaceuticals for the POCIS versus Chemcatcher^®^ was lower (1.84x) than would be expected on the basis of the ratio of active sampling areas (3.01x) of the two devices. The reasons for this difference are hard to determine; one explanation may be due to the sorbent material in the POCIS moving slightly between the PES membranes when the device was deployed in the vertical plane in the deployment cage (Fig. S6). This would decrease the active sampling area of the POCIS and thereby reduce uptake of the analytes (Mills et al. 2014).

An analysis of variance (three-way ANOVA) of the 68 pharmaceuticals identified in the sampler deployments at the four sites was undertaken for the log_10_ integrated peak areas (Table S7). A log_10_ transformation was used because it improved the variance stability greatly over the untransformed data (Fig. S10). The treatments were pharmaceutical, site and sampler. Every interaction term was included. All of the effects and interaction terms were significant. A multiple comparison of the site means using a Bonferroni probability test (Table S8) showed that there were small but statistically significant differences between the overall site means. Site WWTP B2 (308,000) was different from all other sites, whilst WWTP B1 (319,000) and WWTP C (320,000) were not significantly different and WWTP A (414,000) was different from the other three sites. The differences between the overall site means for all of the compounds and both sampler types were relatively small compared with the differences between samplers (Chemcatcher^®^ (218,000) and POCIS (523,000)).

An analysis of variance (two-way ANOVA) of the 68 pharmaceuticals identified in the sampler co-deployments at WWTP B site was undertaken for the log_10_ integrated peak areas for each sampler type (Tables S9 and S10). Sample repeat and compound were selected as factors. An interaction between sample repeat and compound was also calculated. There was no significant difference between the two parallel deployments for Chemcatcher^®^*F*_1,274_ = 0.267), and the interaction term was not significant (*F*_66,274_ > 0.999). However, for POCIS there was a significant (*F*_1,274_ = 0.013) difference between the parallel deployments, but again no significant (*F*_66,274_ > 0.999) interaction. The differences between the two deployments for POCIS at WWTP B were much smaller than those between WWTP B and the other sites. These observations may be attributed to the movement of sorbent material within the POCIS during deployments.

## Conclusions

The two samplers behaved similarly and sequestered the same range of pharmaceutical compounds that were present in the final effluent at three different WWTP. However, there were substantial losses of sorbent material from the POCIS during deployment and the subsequent analytical steps. These losses are often ignored by end-users of the device, but a recovery factor should be considered in the future to improve the overall reliability of the resultant data. Our data showed the uptake ratio between the two devices was lower than that predicted based solely on the difference between their active sampling areas. The most likely contributory factor was the movement of sorbent material within the POCIS during deployment in the vertical plane throughout the field trials significantly reducing the theoretically available surface area for compound adsorption. To minimise this movement it may be beneficial for end-users to deploy the device horizontally in the deployment cage. When comparing uptake rates (*R*_*s*_ values) between the two devices and where the POCIS has been deployed in the vertical plane, we recommend that the ratio of 1.84 is used as a comparative factor in the future by end-users.

## Electronic supplementary material

ESM 1 (DOCX 4.23 mb)

## References

[CR1] Abdallah MA, Nguyen KH, Ebele AJ, Atia NN, Ali HRH, Harrad S (2019). A single run, rapid polarity switching method for determination of 30 pharmaceuticals and personal care products in waste water using Q-Exactive Orbitrap high resolution accurate mass spectrometry. J Chromatogr A.

[CR2] Acena J, Stampachiacchiere S, Perez S, Barcelo D (2015). Advances in liquid chromatography-high-resolution mass spectrometry for quantitative and qualitative environmental analysis. Anal Bioanal Chem.

[CR3] Alvarez DA, Petty JD, Huckins JN, Jones-Lepp TL, Getting DT, Goddard JP, Manahan SE (2004). Development of a passive, in situ, integrative sampler for hydrophilic organic contaminants in aquatic environments. Environ Toxicol Chem.

[CR4] Alvarez DA, Shappell NW, Billey LO, Bermudez DS, Wilson VS, Kolpin DW, Perkins SD, Evans N, Foreman WT, Gray JL, Shipitalo MJ, Meyer MT (2013). Bioassay of estrogenicity and chemical analyses of estrogens in streams across the United States associated with livestock operations. Water Res.

[CR5] Archer E, Petrie B, Kasprzyk-Hordern B, Wolfaardt GM (2017). The fate of pharmaceuticals and personal care products (PPCPs), endocrine disrupting contaminants (EDCs), metabolites and illicit drugs in a WWTW and environmental waters. Chemosphere.

[CR6] Baz-Lomba JA, Harman C, Reid M, Thomas KV (2017). Passive sampling of wastewater as a tool for the long-term monitoring of community exposure: illicit and prescription drug trends as a proof of concept. Water Res.

[CR7] Booij K, Vrana B, & Huckins JN (2007). Theory, modeling and calibration of passive samplers used in water monitoring. In R. Greenwood, G. A. Mills, & B. Vrana (Eds.), Passive sampling techniques in environmental monitoring (pp. 146–169): Elsevier

[CR8] Buzier R, Guibal R, Lissalde S, Guibaud G (2019). Limitation of flow effect on passive sampling accuracy using POCIS with the PRC approach or o-DGT: a pilot-scale evaluation for pharmaceutical compounds. Chemosphere.

[CR9] Castle GD, Mills GA, Bakir A, Gravell A, Schumacher M, Snow K, Fones GR (2018). Measuring metaldehyde in surface waters in the UK using two monitoring approaches. Environ Sci-Proc Imp.

[CR10] Castle GD, Mills GA, Bakir A, Gravell A, Schumacher M, Townsend I, Jones L, Greenwood R, Knott S, Fones GR (2018b) Calibration and field evaluation of the Chemcatcher^®^ passive sampler for monitoring metaldehyde in surface water. Talanta 179:57–63. 10.1016/j.talanta.2017.10.053 10.1016/j.talanta.2017.10.053 29310277

[CR11] Castle GD, Mills GA, Gravell A, Leggatt A, Stubbs J, Davis R, Fones GR (2019). Comparison of different monitoring methods for the measurement of metaldehyde in surface waters. Environ Monit Assess.

[CR12] Challis JK, Hanson ML, Wong CS (2016). Development and calibration of an organic-diffusive gradients in thin films aquatic passive sampler for a diverse suite of polar organic contaminants. Anal Chem.

[CR13] Challis JK, Stroski KM, Luong KH, Hanson ML, Wong CS (2018). Field evaluation and in situ stress testing of the organic-diffusive gradients in thin-films passive sampler. Environ Sci Technol.

[CR14] Charriau A, Lissalde S, Poulier G, Mazzella N, Buzier R, Guibaud G (2016) Overview of the Chemcatcher^®^ for the passive sampling of various pollutants in aquatic environments part a: principles, calibration, preparation and analysis of the sampler. Talanta 148:556–571. 10.1016/j.talanta.2015.06.064 10.1016/j.talanta.2015.06.064 26653485

[CR15] Cornbleet PJ, Gochman N (1979). Incorrect least-squares regression coefficients in method-comparison analysis. Clin Chem.

[CR16] Creusot N, Ait-Aissa S, Tapie N, Pardon P, Brion F, Sanchez W (2014). Identification of synthetic steroids in river water downstream from pharmaceutical manufacture discharges based on a bioanalytical approach and passive sampling. Environ Sci Technol.

[CR17] Daughton CG (2016). Pharmaceuticals and the environment (PiE): evolution and impact of the published literature revealed by bibliometric analysis. Sci Total Environ.

[CR18] Dong J, Fan H, Sui D, Li L, Sun T (2014). Sampling 4-chlorophenol in water by DGT technique with molecularly imprinted polymer as binding agent and nylon membrane as diffusive layer. Anal Chim Acta.

[CR19] EC [European Commission] (2000). Directive 2000/60/EC of the European Parliament and of the Council of 23rd October 2000 establishing a framework for community action in the field of water policy. Off. J. Eur. Communities, (L327/1) (European Commission, Brussels)

[CR20] Fauvelle V, Mazzella N, Belles A, Moreira A, Allan IJ, Budzinski H (2014). Optimization of the polar organic chemical integrative sampler for the sampling of acidic and polar herbicides. Anal Bioanal Chem.

[CR21] García-Córcoles MT, Rodríguez-Gómez R, de Alarcón-Gómez B, Çipa M, Martín-Pozo L, Kauffmann JM, Zafra-Gómez A (2019). Chromatographic methods for the determination of emerging contaminants in natural water and wastewater samples: a review. Crit Rev Anal Chem.

[CR22] Godlewska K, Stepnowski P, & Paszkiewicz M (2019). Application of the polar organic chemical integrative sampler for isolation of environmental micropollutants - a review. Crit Rev Anal Chem 1-28, doi:10.1080/10408347.2019.1565983 10.1080/10408347.2019.1565983 31204504

[CR23] Gravell A (2017). Better ‘tools’ for investigative monitoring under the water framework directive. PhD thesis, University of Portsmouth, Portsmouth, UK

[CR24] Guibal R, Lissalde S, Charriau A, Guibaud G (2015). Improvement of POCIS ability to quantify pesticides in natural water by reducing polyethylene glycol matrix effects from polyethersulfone membranes. Talanta.

[CR25] Guibal R, Buzier R, Charriau A, Lissalde S, Guibaud G (2017). Passive sampling of anionic pesticides using the diffusive gradients in thin films technique (DGT). Anal Chim Acta.

[CR26] Guibal R, Lissalde S, Brizard Y, Guibaud G (2018). Semi-continuous pharmaceutical and human tracer monitoring by POCIS sampling at the watershed-scale in an agricultural rural headwater river. J Hazard Mater.

[CR27] Harman C, Allan IJ, Vermeirssen ELM (2012). Calibration and use of the polar organic chemical integrative sampler-a critical review. Environ Toxicol Chem.

[CR28] Hughes SR, Kay P, Brown LE (2013). Global synthesis and critical evaluation of pharmaceutical data sets collected from river systems. Environ Sci Technol.

[CR29] Kosma CI, Lambropoulou DA, Albanis TA (2016). Analysis, occurrence, fate and risks of proton pump inhibitors, their metabolites and transformation products in aquatic environment: a review. Sci Total Environ.

[CR30] Lissalde S, Charriau A, Poulier G, Mazzella N, Buzier R, Guibaud G (2016) Overview of the Chemcatcher^®^ for the passive sampling of various pollutants in aquatic environments part B: field handling and environmental applications for the monitoring of pollutants and their biological effects. Talanta 148:572–582. 10.1016/j.talanta.2015.06.076 10.1016/j.talanta.2015.06.076 26653486

[CR31] Lohmann R, Booij K, Smedes F, Vrana B (2012). Use of passive sampling devices for monitoring and compliance checking of POP concentrations in water. Environ Sci Pollut Res.

[CR32] Mills GA, Gravell A, Vrana B, Harman C, Budzinski H, Mazzella N, Ocelka T (2014). Measurement of environmental pollutants using passive sampling devices - an updated commentary on the current state of the art. Environ Sci-Proc Imp.

[CR33] Morin N, Miege C, Randon J, Coquery M (2012). Chemical calibration, performance, validation and applications of the polar organic chemical integrative sampler (POCIS) in aquatic environments. TrAC-Trend Anal Chem.

[CR34] Morin N, Camilleri J, Cren-Olive C, Coquery M, Miege C (2013). Determination of uptake kinetics and sampling rates for 56 organic micropollutants using “pharmaceutical” POCIS. Talanta.

[CR35] Petrie B, McAdam EJ, Scrimshaw MD, Lester JN, Cartmell E (2013). Fate of drugs during wastewater treatment. TrAC-Trend Anal Chem.

[CR36] Petrie B, Barden R, Kasprzyk-Hordern B (2015). A review on emerging contaminants in wastewaters and the environment: current knowledge, understudied areas and recommendations for future monitoring. Water Res.

[CR37] Petrie B, Gravell A, Mills GA, Youdan J, Barden R, Kasprzyk-Hordern B (2016). In situ calibration of a new Chemcatcher configuration for the determination of polar organic micropollutants in wastewater effluent. Environ Sci Technol.

[CR38] Petty JD, Huckins JN, Alvarez DA, Brumbaugh WG, Cranor WL, Gale RW, Rastall AC, Jones-Lepp TL, Leiker TJ, Rostad CE, Furlong ET (2004). A holistic passive integrative sampling approach for assessing the presence and potential impacts of waterborne environmental contaminants. Chemosphere.

[CR39] Pinasseau L, Wiest L, Fildier A, Volatier L, Fones GR, Mills GA, Mermillod-Blondin F, Vulliet E (2019). Use of passive sampling and high resolution mass spectrometry using a suspect screening approach to characterise emerging pollutants in contaminated groundwater and runoff. Sci Total Environ.

[CR40] Rimayi C, Chimuka L, Gravell A, Fones GR, Mills GA (2019) Use of the Chemcatcher^®^ passive sampler and time-of-flight mass spectrometry to screen for emerging pollutants in rivers in Gauteng Province of South Africa. Environ Monit Assess 191(6):388. 10.1007/s10661-019-7515-z 10.1007/s10661-019-7515-z PMC6529598 31115701

[CR41] Seen A, Bizeau O, Sadler L, Jordan T, Nichols D (2014). Assessment of Envi-Carb™ as a passive sampler binding phase for acid herbicides without pH adjustment. Chemosphere.

[CR42] Taylor AC, Fones GR, Vrana B, Mills GA (2019). Applications for passive sampling of hydrophobic organic contaminants in water - a review. Crit Rev Anal Chem.

[CR43] Townsend I, Jones L, Broom M, Gravell A, Schumacher M, Fones GR, Greenwood R, Mills GA (2018) Calibration and application of the Chemcatcher^®^ passive sampler for monitoring acidic herbicides in the River Exe, UK catchment. Environ Sci Pollut Res 25(25):25130–25142. 10.1007/s11356-018-2556-3 10.1007/s11356-018-2556-3 PMC6133114 29943243

[CR44] Urik J, Vrana B (2019). An improved design of a passive sampler for polar organic compounds based on diffusion in agarose hydrogel. Environ Sci Pollut Res.

[CR45] Vermeirssen ELM, Dietschweiler C, Escher BI, van der Voet J, Hollender J (2012). Transfer kinetics of polar organic compounds over polyethersulfone membranes in the passive samplers POCIS and Chemcatcher. Environ Sci Technol.

[CR46] Vrana B, Mills GA, Allan IJ, Dominiak E, Svensson K, Knutsson J (2005). Passive sampling techniques for monitoring pollutants in water. TrAC-Trend Anal Chem.

[CR47] Vrana B, Mills GA, Kotterman M, Leonards P, Booij K, Greenwood R (2007). Modelling and field application of the Chemcatcher passive sampler calibration data for the monitoring of hydrophobic organic pollutants in water. Environ Pollut.

[CR48] Zheng J-L, Guan D-X, Luo J, Zhang H, Davison W, Cui X-Y, Wang LH, Ma LQ (2015). Activated charcoal based diffusive gradients in thin films for in situ monitoring of bisphenols in waters. Anal Chem.

