# Supplementary material for: Detection of pharmaceuticals in wastewater effluents—a comparison of the performance of Chemcatcher® and polar organic compound integrative sampler
Source: Environ Sci Pollut Res Int. 2020 May 13;27(22):27995–8005. doi: 10.1007/s11356-020-09077-5 (PMC7334249; doi:10.1007/s11356-020-09077-5)
Supplement: Supplementary file 1 — (DOCX 4.23 mb) [file 11356_2020_9077_MOESM1_ESM.docx]

**Supplementary Material**

**Detection of pharmaceuticals in wastewater effluents – a comparison of the performance of Chemcatcher® and Polar Organic Compound Integrative Sampler**

Anthony Gravell^a^, Gary R. Fones^b*^, Richard Greenwood^c^ and Graham A. Mills^d^

^a^Natural Resources Wales, Faraday Building, Swansea University, Singleton Campus, Swansea, SA2 8PP, UK

^b^School of Earth and Environmental Sciences, University of Portsmouth, Burnaby Road, Portsmouth, PO1 3QL, UK

^c^School of Biological Sciences, University of Portsmouth, King Henry Building, King Henry I Street, Portsmouth, PO1 2DY, UK

^d^School of Pharmacy and Biomedical Sciences, University of Portsmouth, White Swan Road, Portsmouth, PO1 2DT, UK

^*^Corresponding author: Gary Fones: [gary.fones@port.ac.uk](mailto:gary.fones@port.ac.uk); +44(0)23 9284 2252

**Identification of pharmaceutical compounds and development of LC/Q-ToF-MS database**

In order to curate a pharmaceutical compound database library to be used with the LC/Q-ToF-MS system, a search (using the Welsh government website: <http://gov.wales/statistics-and-research/prescriptions-dispensed-community/>) was undertaken to identify the most prescribed pharmaceuticals by general medical practitioners (GPs) in Wales in 2014. It was expected that these substances might be present in final effluents and watercourses receiving discharges from wastewater treatment plants (WWTPs).

For practical reasons only individual pharmaceuticals (by chemical name) were included in the data base where the total number of prescriptions exceeded 25,000 that year. This list was further refined by excluding compounds that were not amenable to reversed-phase conditions typically used in LC-MS methods and those exhibiting poor solubility in water or organic solvents. These compounds included sugars, metal-based compounds, vitamins and other dietary supplements, oils, oxidisers, solvents, insulin and common inorganic salts. Pharmaceutical compounds previously analysed at the Natural Resources Wales laboratory (as part of the UK Water Industry Research Chemical Investigation Programme) were also included in the list. This resulted in 164 pharmaceutical compounds for which standards were purchased (5 mg, purity ≥ 95%, Sigma-Aldrich) (Table S1).

**Table S1** List of pharmaceuticals for which reference materials were purchased for Q-TOF-MS analysis

| **Pharmaceutical class** | **Compound determined** | **CAS RN** | **Chemspider ID** | **Systematic (IUPAC) name for compound determined - obtained from PubChem** |
| --- | --- | --- | --- | --- |
| Analgesic | Sumatriptan | 103628-46-2 | 5165 | 1-[3-[2-(dimethylamino)ethyl]-1H-indol-5-yl]-N-methylmethanesulfonamide |
|  | Tramadol | 27203-92-5 | 31105 | (1R,2R)-2-[(dimethylamino)methyl]-1-(3-methoxyphenyl)cyclohexan-1-ol |
| Antacid | Ranitidine | 66357-35-5 | 571454 | (E)-1-N'-[2-[[5-[(dimethylamino)methyl]furan-2-yl]methylsulfanyl]ethyl]-1-N-methyl-2-nitroethene-1,1-diamine |
| Anti-arrhythmic | Amiodarone | 1951-25-3 | 2072 | (2-butyl-1-benzofuran-3-yl)-[4-[2-(diethylamino)ethoxy]-3,5-diiodophenyl]methanone |
|  | Flecainide | 54143-55-4 | 3239 | N-(piperidin-2-ylmethyl)-2,5-bis(2,2,2-trifluoroethoxy)benzamide |
| Anti-asthmatic | Cromolyn | 16110-51-3 | 2779 | 5-[3-(2-carboxy-4-oxochromen-5-yl)oxy-2-hydroxypropoxy]-4-oxochromene-2-carboxylic acid |
|  | Ipratropium (as the cation) | 60205-81-4 | 3615 | [(1S,5R)-8-methyl-8-propan-2-yl-8-azoniabicyclo[3.2.1]octan-3-yl] 3-hydroxy-2-phenylpropanoate |
|  | Salbutamol | 18559-94-9 | 1999 | 4-[2-(tert-butylamino)-1-hydroxyethyl]-2-(hydroxymethyl)phenol |
|  | Salmeterol | 89365-50-4 | 4968 | 2-(hydroxymethyl)-4-[1-hydroxy-2-[6-(4-phenylbutoxy)hexylamino]ethyl]phenol |
| Anti-biotic | Amoxicillin | 26787-78-0 | 31006 | (2S,5R,6R)-6-[[(2R)-2-amino-2-(4-hydroxyphenyl)acetyl]amino]-3,3-dimethyl-7-oxo-4-thia-1-azabicyclo[3.2.0]heptane-2-carboxylic acid |
|  | Cefalexin | 15686-71-2 | 25541 | (6R,7R)-7-[[(2R)-2-amino-2-phenylacetyl]amino]-3-methyl-8-oxo-5-thia-1-azabicyclo[4.2.0]oct-2-ene-2-carboxylic acid |
|  | Chloramphenicol | 56-75-7 | 5744 | 2,2-dichloro-N-[(1R,2R)-1,3-dihydroxy-1-(4-nitrophenyl)propan-2-yl]acetamide |
|  | Chlortetracycline | 57-62-5 | 10469370 | (4S,4aS,5aS,6S,12aR)-7-chloro-4-(dimethylamino)-1,6,10,11,12a-pentahydroxy-6-methyl-3,12-dioxo-4,4a,5,5a-tetrahydrotetracene-2-carboxamide |
|  | Clarithromycin | 81103-11-9 | 4447591 | (3R,4S,5S,6R,7R,9R,11R,12R,13S,14R)-6-[(2S,3R,4S,6R)-4-(dimethylamino)-3-hydroxy-6-methyloxan-2-yl]oxy-14-ethyl-12,13-dihydroxy-4-[(2R,4R,5S,6S)-5-hydroxy-4-methoxy-4,6-dimethyloxan-2-yl]oxy-7-methoxy-3,5,7,9,11,13-hexamethyl-oxacyclotetradecane-2,10-dione |
|  | Doxycycline | 564-25-0 | 10469369 | (4S,4aR,5S,5aR,6R,12aR)-4-(dimethylamino)-1,5,10,11,12a-pentahydroxy-6-methyl-3,12-dioxo-4a,5,5a,6-tetrahydro-4H-tetracene-2-carboxamide |
|  | Erythromycin | 114-07-8 | 12041 | (3R,4S,5S,6R,7R,9R,11R,12R,13S,14R)-6-[(2S,3R,4S,6R)-4-(dimethylamino)-3-hydroxy-6-methyloxan-2-yl]oxy-14-ethyl-7,12,13-trihydroxy-4-[(2R,4R,5S,6S)-5-hydroxy-4-methoxy-4,6-dimethyloxan-2-yl]oxy-3,5,7,9,11,13-hexamethyl-oxacyclotetradecane-2,10-dione |
|  | Nitrofurantoin | 67-20-9 | 5036498 | 1-[(E)-(5-nitrofuran-2-yl)methylideneamino]imidazolidine-2,4-dione |
|  | Nystatin | 34786-70-4 | 10468627 | (1S,3R,4R,7R,9R,11R,15S,16R,17R,18S,19E,21E,25E,27E,29E,31E,33R,35S,36R,37S)-33-[(3-Amino-3,6-dideoxy-beta-D-mannopyranosyl)oxy]-1,3,4,7,9,11,17,37-octahydroxy-15,16,18-trimethyl-13-oxo-14,39-dioxabic yclo[33.3.1]nonatriaconta-19,21,25,27,29,31-hexaene-36-carboxylic acid |
|  | Ofloxacin | 82419-36-1 | 4422 | 9-Fluoro-3-methyl-10-(4-methyl-1-piperazinyl)-7-oxo-2,3-dihydro-7H-[1,4]oxazino[2,3,4-ij]quinoline-6-carboxylic acid |
|  | Oxytetracycline | 79-57-2 | 10482174 | (4S,4aR,5S,5aR,6S,12aR)-4-(dimethylamino)-1,5,6,10,11,12a-hexahydroxy-6-methyl-3,12-dioxo-4,4a,5,5a-tetrahydrotetracene-2-carboxamide |
|  | Penicillin V | 87-08-1 | 6607 | (2S,5R,6R)-3,3-dimethyl-7-oxo-6-[(2-phenoxyacetyl)amino]-4-thia-1-azabicyclo[3.2.0]heptane-2-carboxylic acid |
|  | Trimethoprim | 738-70-5 | 5376 | 5-[(3,4,5-trimethoxyphenyl)methyl]pyrimidine-2,4-diamine **continued** |
| Anti-coagulant | Dipyridamole | 58-32-2 | 2997 | 2-[[2-[bis(2-hydroxyethyl)amino]-4,8-di(piperidin-1-yl)pyrimido[5,4-d]pyrimidin-6-yl]-(2-hydroxyethyl)amino]ethanol |
|  | Warfarin | 81-81-2 | 10442445 | 4-hydroxy-3-(3-oxo-1-phenylbutyl)chromen-2-one |
| Anti-convulsant | Carbamazepine | 298-46-4 | 2457 | Benzo[b][1]benzazepine-11-carboxamide |
|  | Lamotrigine | 84057-84-1 | 3741 | 6-(2,3-dichlorophenyl)-1,2,4-triazine-3,5-diamine |
|  | Phenytoin | 57-41-0 | 1710 | 5,5-diphenylimidazolidine-2,4-dione |
| Anti-depressant | Amitriptyline | 50-48-6 | 2075 | 3-(5,6-dihydrodibenzo[2,1-b:2',1'-f][7]annulen-11-ylidene)-N,N-dimethylpropan-1-amine |
|  | Citalopram | 59729-33-8 | 2669 | 1-[3-(dimethylamino)propyl]-1-(4-fluorophenyl)-3H-2-benzofuran-5-carbonitrile |
|  | Clomipramine | 303-49-1 | 2699 | 3-(2-chloro-5,6-dihydrobenzo[b][1]benzazepin-11-yl)-N,N-dimethylpropan-1-amine |
|  | Dosulepin | 113-53-1 | 4445580 | (3Z)-3-(6H-benzo[c][1]benzothiepin-11-ylidene)-N,N-dimethylpropan-1-amine |
|  | Fluoxetine | 54910-89-3 | 3269 | N-methyl-3-phenyl-3-[4-(trifluoromethyl)phenoxy]propan-1-amine |
|  | Lofepramine | 23047-25-8 | 3810 | 1-(4-chlorophenyl)-2-[3-(5,6-dihydrobenzo[b][1]benzazepin-11-yl)propyl-methylamino]ethanone |
|  | Mirtazapine | 61337-67-5 | 4060 | 2-Methyl-1,2,3,4,10,14b-hexahydropyrazino[2,1-a]pyrido[2,3-c][2]benzazepine |
|  | Paroxetine | 61869-08-7 | 39888 | (3S,4R)-3-(1,3-benzodioxol-5-yloxymethyl)-4-(4-fluorophenyl)piperidine |
|  | Sertraline | 79617-96-2 | 61881 | (1S,4S)-4-(3,4-dichlorophenyl)-N-methyl-1,2,3,4-tetrahydronaphthalen-1-amine |
|  | Trazodone | 19794-93-5 | 5332 | 2-[3-[4-(3-chlorophenyl)piperazin-1-yl]propyl]-[1,2,4]triazolo[4,3-a]pyridin-3-one |
|  | Venlafaxine (Venlaxafine) | 93413-69-5 | 5454 | 1-[2-(dimethylamino)-1-(4-methoxyphenyl)ethyl]cyclohexan-1-ol |
| Anti-diabetic | Gliclazide | 21187-98-4 | 3356 | 1-(3,3a,4,5,6,6a-hexahydro-1H-cyclopenta[c]pyrrol-2-yl)-3-(4-methylphenyl)sulfonylurea |
| Anti-diarrhoeal | Loperamide | 53179-11-6 | 3818 | 4-[4-(4-chlorophenyl)-4-hydroxypiperidin-1-yl]-N,N-dimethyl-2,2-diphenylbutanamide |
| Anti-emetic | Cyclizine / Marzine | 82-92-8 | 6470 | 1-benzhydryl-4-methylpiperazine |
|  | Metoclopramide | 364-62-5 | 4024 | 4-amino-5-chloro-N-[2-(diethylamino)ethyl]-2-methoxybenzamide |
|  | Scopolamine | 51-34-3 | 10194106 | (1R,2R,4S,5S,7s)-9-Methyl-3-oxa-9-azatricyclo[3.3.1.0~2,4~]non-7-yl (2S)-3-hydroxy-2-phenylpropanoate |
| Anti-estrogen | Tamoxifen | 10540-29-1 | 2015313 | 2-[4-[(Z)-1,2-diphenylbut-1-enyl]phenoxy]-N,N-dimethylethanamine |
| Anti-fungal | Clotrimazole | 23593-75-1 | 2710 | 1-[(2-chlorophenyl)-diphenylmethyl]imidazole |
|  | Fluconazole | 86386-73-4 | 3248 | 2-(2,4-difluorophenyl)-1,3-bis(1,2,4-triazol-1-yl)propan-2-ol |
|  | Ketoconazole | 65277-42-1 | 401695 | 1-[4-[4-[[(2S,4R)-2-(2,4-dichlorophenyl)-2-(imidazol-1-ylmethyl)-1,3-dioxolan-4-yl]methoxy]phenyl]piperazin-1-yl]ethanone |
|  | Miconazole | 22916-47-8 | 4044 | 1-[2-(2,4-dichlorophenyl)-2-[(2,4-dichlorophenyl)methoxy]ethyl]imidazole |
|  | Terbinafine | 91161-71-6 | 1266005 | (E)-N,6,6-trimethyl-N-(naphthalen-1-ylmethyl)hept-2-en-4-yn-1-amine |
| Anti-glaucoma | Latanoprost | 130209-82-4 | 4470740 | Propan-2-yl (Z)-7-[(1R,2R,3R,5S)-3,5-dihydroxy-2-[(3R)-3-hydroxy-5-phenylpentyl]cyclopentyl]hept-5-enoate **continued** |
| Anti-histamine | Cetirizine | 83881-51-0 | 2577 | 2-[2-[4-[(4-chlorophenyl)-phenylmethyl]piperazin-1-yl]ethoxy]acetic acid |
|  | Chlorpheniramine | 132-22-9 | 2624 | 3-(4-chlorophenyl)-N,N-dimethyl-3-pyridin-2-ylpropan-1-amine |
|  | Fexofenadine | 83799-24-0 | 3231 | 2-[4-[1-hydroxy-4-[4-[hydroxy(diphenyl)methyl]piperidin-1-yl]butyl]phenyl]-2-methylpropanoic acid |
|  | Hydroxyzine | 68-88-2 | 3531 | 2-[2-[4-[(4-chlorophenyl)-phenylmethyl]piperazin-1-yl]ethoxy]ethanol |
|  | Loratadine | 79794-75-5 | 3820 | Ethyl 4-(8-chloro-5,6-dihydrobenzo[1,2]cyclohepta[2,4-b]pyridin-11-ylidene)piperidine-1-carboxylate |
|  | Promethazine | 60-87-7 | 4758 | N,N-dimethyl-1-phenothiazin-10-ylpropan-2-amine |
|  | Cinnarizine | 298-57-7 | 1264793 | 1-benzhydryl-4-[(E)-3-phenylprop-2-enyl]piperazine |
| Anti-hypertensive | Alfuzosin | 81403-80-7 | 2008 | N-[3-[(4-amino-6,7-dimethoxyquinazolin-2-yl)-methylamino]propyl]oxolane-2-carboxamide |
|  | Amlodipine | 88150-42-9 | 2077 | 3-O-ethyl 5-O-methyl 2-(2-aminoethoxymethyl)-4-(2-chlorophenyl)-6-methyl-1,4-dihydropyridine-3,5-dicarboxylate |
|  | Atenolol | 50-78-2 | 2162 | 2-[4-[2-hydroxy-3-(propan-2-ylamino)propoxy]phenyl]acetamide |
|  | Bisoprolol | 66722-44-9 | 2312 | 1-(propan-2-ylamino)-3-[4-(2-propan-2-yloxyethoxymethyl)phenoxy]propan-2-ol |
|  | Candesartan | 139481-59-7 | 2445 | 2-ethoxy-3-[[4-[2-(2H-tetrazol-5-yl)phenyl]phenyl]methyl]benzimidazole-4-carboxylic acid |
|  | Carvedilol | 72956-09-3 | 2487 | 1-(9H-carbazol-4-yloxy)-3-[2-(2-methoxyphenoxy)ethylamino]propan-2-ol |
|  | Celiprolol | 56980-93-9 | 2563 | 3-[3-acetyl-4-[3-(tert-butylamino)-2-hydroxypropoxy]phenyl]-1,1-diethylurea |
|  | Clonidine | 4205-90-7 | 2701 | N-(2,6-dichlorophenyl)-4,5-dihydro-1H-imidazol-2-amine |
|  | Diltiazem-Cis | 42399-41-7 | 35850 | [(2S,3S)-5-[2-(dimethylamino)ethyl]-2-(4-methoxyphenyl)-4-oxo-2,3-dihydro-1,5-benzothiazepin-3-yl] acetate |
|  | Doxazosin | 74191-85-8 | 3045 | [4-(4-amino-6,7-dimethoxyquinazolin-2-yl)piperazin-1-yl]-(2,3-dihydro-1,4-benzodioxin-3-yl)methanone |
|  | Enalapril | 75847-73-3 | 4534998 | (2S)-1-[(2S)-2-[[(2S)-1-ethoxy-1-oxo-4-phenylbutan-2-yl]amino]propanoyl]pyrrolidine-2-carboxylic acid |
|  | Felodipine | 72509-76-3 | 3216 | 5-O-ethyl 3-O-methyl 4-(2,3-dichlorophenyl)-2,6-dimethyl-1,4-dihydropyridine-3,5-dicarboxylate |
|  | Irbesartan | 138402-11-6 | 3618 | 2-butyl-3-[[4-[2-(2H-tetrazol-5-yl)phenyl]phenyl]methyl]-1,3-diazaspiro[4.4]non-1-en-4-one |
|  | Labetalol | 36894-69-6 | 3734 | 2-hydroxy-5-[1-hydroxy-2-(4-phenylbutan-2-ylamino)ethyl]benzamide |
|  | Lisinopril | 76547-98-3 | 4514933 | (2S)-1-[(2S)-6-amino-2-[[(1S)-1-carboxy-3-phenylpropyl]amino]hexanoyl]pyrrolidine-2-carboxylic acid |
|  | Losartan | 114798-26-4 | 3824 | [2-butyl-5-chloro-3-[[4-[2-(2H-tetrazol-5-yl)phenyl]phenyl]methyl]imidazol-4-yl]methanol |
|  | Metoprolol | 37350-58-6 | 4027 | 1-[4-(2-methoxyethyl)phenoxy]-3-(propan-2-ylamino)propan-2-ol |
|  | Moxonidine | 75438-57-2 | 4645 | 4-chloro-N-(4,5-dihydro-1H-imidazol-2-yl)-6-methoxy-2-methylpyrimidin-5-amine |
|  | Nebivolol | 99200-09-6 | 64421 | 1-(6-fluoro-3,4-dihydro-2H-chromen-2-yl)-2-[[2-(6-fluoro-3,4-dihydro-2H-chromen-2-yl)-2-hydroxyethyl]amino]ethanol |
|  | Nifedipine | 21829-25-4 | 4330 | Dimethyl 2,6-dimethyl-4-(2-nitrophenyl)-1,4-dihydropyridine-3,5-dicarboxylate |
|  | Oxprenolol | 6452-71-7 | 4470 | 1-(propan-2-ylamino)-3-(2-prop-2-enoxyphenoxy)propan-2-ol **continued** |
|  | Perindopril | 82834-16-0 | 96956 | (2S,3aS,7aS)-1-[(2S)-2-[[(2S)-1-ethoxy-1-oxopentan-2-yl]amino]propanoyl]-2,3,3a,4,5,6,7,7a-octahydroindole-2-carboxylic acid |
|  | Propranolol | 525-66-6 | 4777 | 1-naphthalen-1-yloxy-3-(propan-2-ylamino)propan-2-ol |
|  | Ramipril | 87333-19-5 | 4514937 | (2S,3aS,6aS)-1-[(2S)-2-[[(2S)-1-ethoxy-1-oxo-4-phenylbutan-2-yl]amino]propanoyl]-3,3a,4,5,6,6a-hexahydro-2H-cyclopenta[b]pyrrole-2-carboxylic acid |
|  | Sotalol | 3930-20-9 | 5063 | N-[4-[1-hydroxy-2-(propan-2-ylamino)ethyl]phenyl]methanesulfonamide |
|  | Telmisartan | 144701-48-4 | 59391 | 2-[4-[[4-methyl-6-(1-methylbenzimidazol-2-yl)-2-propylbenzimidazol-1-yl]methyl]phenyl]benzoic acid |
|  | Timolol | 26839-75-8 | 31013 | (2S)-1-(tert-butylamino)-3-[(4-morpholin-4-yl-1,2,5-thiadiazol-3-yl)oxy]propan-2-ol |
|  | Valsartan | 137862-53-4 | 54833 | (2S)-3-methyl-2-[pentanoyl-[[4-[2-(2H-tetrazol-5-yl)phenyl]phenyl]methyl]amino]butanoic acid |
|  | Verapamil | 52-53-9 | 2425 | 2-(3,4-dimethoxyphenyl)-5-[2-(3,4-dimethoxyphenyl)ethyl-methylamino]-2-propan-2-ylpentanenitrile |
| Anti-infective | Chlorhexidine | 55-56-1 | 2612 | (1E)-2-[6-[[amino-[(E)-[amino-(4-chloroanilino)methylidene]amino]methylidene]amino]hexyl]-1-[amino-(4-chloroanilino)methylidene]guanidine |
| Anti-malarial | Quinine | 130-95-0 | 84989 | (R)-[(2S,4S,5R)-5-ethenyl-1-azabicyclo[2.2.2]octan-2-yl]-(6-methoxyquinolin-4-yl)methanol |
| Anti-neoplastic | Anastrozole | 120511-73-1 | 2102 | 2-[3-(2-cyanopropan-2-yl)-5-(1,2,4-triazol-1-ylmethyl)phenyl]-2-methylpropanenitrile |
| Anti-obesity | Orlistat | 96829-58-2 | 2298564 | [(2S)-1-[(2S,3S)-3-hexyl-4-oxooxetan-2-yl]tridecan-2-yl] (2S)-2-formamido-4-methylpentanoate |
| Anti-platelet | Clopidogrel | 113665-84-2 | 54632 | Methyl (2S)-2-(2-chlorophenyl)-2-(6,7-dihydro-4H-thieno[3,2-c]pyridin-5-yl)acetate |
| Anti-psychotic | Amisulpride | 71675-85-9 | 2074 | 4-amino-N-[(1-ethylpyrrolidin-2-yl)methyl]-5-ethylsulfonyl-2-methoxybenzamide |
|  | Chlorpromazine | 50-53-3 | 2625 | 3-(2-chlorophenothiazin-10-yl)-N,N-dimethylpropan-1-amine |
|  | Haloperidol | 52-86-8 | 3438 | 4-[4-(4-chlorophenyl)-4-hydroxypiperidin-1-yl]-1-(4-fluorophenyl)butan-1-one |
|  | Risperidone | 106266-06-2 | 4895 | 3-[2-[4-(6-fluoro-1,2-benzoxazol-3-yl)piperidin-1-yl]ethyl]-2-methyl-6,7,8,9-tetrahydropyrido[1,2-a]pyrimidin-4-one |
| Anti-rheumatic / Anti-malarial | Hydroxychloroquine | 118-42-3 | 3526 | 2-[4-[(7-chloroquinolin-4-yl)amino]pentyl-ethylamino]ethanol |
| Anti-malarial | Sulfasalazine | 599-79-1 | 10481900 | (3Z)-6-oxo-3-[[4-(pyridin-2-ylsulfamoyl)phenyl]hydrazinylidene]cyclohexa-1,4-diene-1-carboxylic acid |
| Anti-spasmodic | Mebeverine | 630-20-3 | 3891 | 4-[ethyl-[1-(4-methoxyphenyl)propan-2-yl]amino]butyl 3,4-dimethoxybenzoate |
|  | Oxybutynin | 5633-20-5 | 4473 | 4-(diethylamino)but-2-ynyl 2-cyclohexyl-2-hydroxy-2-phenylacetate |
|  | Procyclidine | 77-37-2 | 4750 | 1-cyclohexyl-1-phenyl-3-pyrrolidin-1-ylpropan-1-ol |
| Bronchodilator | Terbutaline | 23031-25-6 | 5210 | 5-[2-(tert-butylamino)-1-hydroxyethyl]benzene-1,3-diol |
|  | Theophylline | 58-55-9 | 2068 | 1,3-dimethyl-7H-purine-2,6-dione |
| Cardiotonic drug | Digoxin | 20830-75-5 | 2006532 | 3-[(3S,5R,8R,9S,10S,12R,13S,14S,17R)-3-[(2R,4S,5S,6R)-5-[(2S,4S,5S,6R)-5-[(2S,4S,5S,6R)-4,5-dihydroxy-6-methyloxan-2-yl]oxy-4-hydroxy-6-methyloxan-2-yl]oxy-4-hydroxy-6-methyloxan-2-yl]oxy-12,14-dihydroxy-10,13-dimethyl-1,2,3,4,5,6,7,8,9,11,12,15,16,17-tetradecahydrocyclopenta[a]phenanthren-17-yl]-2H-furan-5-one |
| Cholinergic antagonist | Alverine | 150-59-4 | 3550 | N-ethyl-3-phenyl-N-(3-phenylpropyl)propan-1-amine **continued** |
| Corticosteroid | Betamethasone | 378-44-9 | 9399 | (4aR,4bS,5S,6aS,6bS,9aR,10aS,10bS)-6b-Glycoloyl-5-hydroxy-4a,6a-dimethyl-8-propyl-4a,4b,5,6,6a,6b,9a,10,10a,10b,11,12-dodecahydro-2H-naphtho[2',1':4,5]indeno[1,2-d][1,3]dioxol-2-one |
|  | Betamethasone-17-valerate | 2152-44-5 | 15673 | [(8S,9R,10S,11S,13S,14S,16S,17R)-9-fluoro-11-hydroxy-17-(2-hydroxyacetyl)-10,13,16-trimethyl-3-oxo-6,7,8,11,12,14,15,16-octahydrocyclopenta[a]phenanthren-17-yl] pentanoate |
| Diuretic | Amiloride | 2609-46-3 | 15403 | 3,5-diamino-6-chloro-N-(diaminomethylidene)pyrazine-2-carboxamide |
|  | Bendroflumethiazide | 73-48-3 | 2225 | 3-benzyl-1,1-dioxo-6-(trifluoromethyl)-3,4-dihydro-2H-1,2,4-benzothiadiazine-7-sulfonamide |
|  | Bumetanide | 28395-03-1 | 2377 | 3-(butylamino)-4-phenoxy-5-sulfamoylbenzoic acid |
|  | Furosemide | 54-31-9 | 3322 | 4-chloro-2-(furan-2-ylmethylamino)-5-sulfamoylbenzoic acid |
|  | Hydrochlorothiazide | 58-93-5 | 3513 | 6-chloro-1,1-dioxo-3,4-dihydro-2H-1,2,4-benzothiadiazine-7-sulfonamide |
|  | Indapamide | 26807-65-8 | 3574 | 4-chloro-N-(2-methyl-2,3-dihydroindol-1-yl)-3-sulfamoylbenzamide |
|  | Spironolactone | 52-01-7 | 5628 | S-[(7R,8R,9S,10R,13S,14S,17R)-10,13-dimethyl-3,5'-dioxospiro[2,6,7,8,9,11,12,14,15,16-decahydro-1H-cyclopenta[a]phenanthrene-17,2'-oxolane]-7-yl] ethanethioate |
| Dopamine agonist | Ropinirole | 91374-21-9 | 4916 | 4-[2-(dipropylamino)ethyl]-1,3-dihydroindol-2-one |
| Laxative | Bisacodyl | 603-50-9 | 2299 | [4-[(4-acetyloxyphenyl)-pyridin-2-ylmethyl]phenyl] acetate |
|  | Dioctyl sulfosuccinate | 10041-19-7 | 10862 | 1,4-bis(2-ethylhexoxy)-1,4-dioxobutane-2-sulfonate |
| Lipid regulator | Atorvastatin | 134523-00-5 | 54810 | (3R,5R)-7-[2-(4-fluorophenyl)-3-phenyl-4-(phenylcarbamoyl)-5-propan-2-ylpyrrol-1-yl]-3,5-dihydroxyheptanoic acid |
|  | Bezafibrate | 41859-67-0 | 35728 | 2-[4-[2-[(4-chlorobenzoyl)amino]ethyl]phenoxy]-2-methylpropanoic acid |
|  | Fenofibrate | 49562-28-9 | 3222 | Propan-2-yl 2-[4-(4-chlorobenzoyl)phenoxy]-2-methylpropanoate |
|  | Pravastatin | 81093-37-0 | 49398 | (3R,5R)-7-[(1S,2S,6S,8S,8aR)-6-hydroxy-2-methyl-8-[(2S)-2-methylbutanoyl]oxy-1,2,6,7,8,8a-hexahydronaphthalen-1-yl]-3,5-dihydroxyheptanoic acid |
|  | Simvastatin | 79902-63-9 | 49179 | [(1S,3R,7S,8S,8aR)-8-[2-[(2R,4R)-4-hydroxy-6-oxooxan-2-yl]ethyl]-3,7-dimethyl-1,2,3,7,8,8a-hexahydronaphthalen-1-yl] 2,2-dimethylbutanoate |
| Local anasthetic | Lidocaine | 137-58-6 | 3548 | 2-(diethylamino)-N-(2,6-dimethylphenyl)acetamide |
| Nicotinic antagonist | Varenicline | 249296-44-4 | 4470510 | (1R,12S)-5,8,14-Triazatetracyclo[10.3.1.0~2,11~.0~4,9~]hexadeca-2,4,6,8,10-pentaene |
| Nootropic agent | Donepezil | 120014-06-4 | 3040 | 2-[(1-benzylpiperidin-4-yl)methyl]-5,6-dimethoxy-2,3-dihydroinden-1-one |
| NSAID | Acetaminophen | 103-90-2 | 1906 | N-(4-Hydroxyphenyl)acetamide |
|  | Acetylsalicylic acid | 50-78-2 | 2157 | 2-Acetoxybenzoic acid |
|  | Benzindamine | 642-72-8 | 12036 | 3-(1-benzylindazol-3-yl)oxy-N,N-dimethylpropan-1-amine |
|  | Celecoxib | 169590-42-5 | 2562 | 4-[5-(4-methylphenyl)-3-(trifluoromethyl)pyrazol-1-yl]benzenesulfonamide |
|  | Diclofenac | 15307-86-5 | 2925 | 2-[2-(2,6-dichloroanilino)phenyl]acetic acid |
|  | Ibuprofen | 15687-27-1 | 3544 | 2-[4-(2-methylpropyl)phenyl]propanoic acid **continued** |
|  | Ketoprofen | 22071-15-4 | 3693 | 2-(3-benzoylphenyl)propanoic acid |
|  | Mefenamic acid | 61-68-7 | 3904 | 2-(2,3-dimethylanilino)benzoic acid |
|  | Meloxicam | 71125-38-7 | 10442740 | 4-hydroxy-2-methyl-N-(5-methyl-1,3-thiazol-2-yl)-1,1-dioxo-1,2-benzothiazine-3-carboxamide |
|  | Naproxen | 22204-53-1 | 137720 | (2S)-2-(6-methoxynaphthalen-2-yl)propanoic acid |
|  | Piroxicam | 36322-90-4 | 10442653 | 4-Hydroxy-2-methyl-N-(2-pyridinyl)-2H-1,2-benzothiazine-3-carboximidic acid 1,1-dioxide |
|  | Salicylic acid | 69-72-7 | 331 | 2-hydroxybenzoic acid |
| PDE5 inhibitor | Sildenafil | 139755-83-2 | 5023 | 5-[2-ethoxy-5-(4-methylpiperazin-1-yl)sulfonylphenyl]-1-methyl-3-propyl-4H-pyrazolo[4,3-d]pyrimidin-7-one |
| Proton pump inhibitor | Lansoprazole | 103577-45-3 | 3746 | 2-[[3-methyl-4-(2,2,2-trifluoroethoxy)pyridin-2-yl]methylsulfinyl]-1H-benzimidazole |
|  | Omeprazole | 73590-58-6 | 4433 | 6-methoxy-2-[(4-methoxy-3,5-dimethylpyridin-2-yl)methylsulfinyl]-1H-benzimidazole |
|  | Pantoprazole | 102625-70-7 | 4517 | 6-(difluoromethoxy)-2-[(3,4-dimethoxypyridin-2-yl)methylsulfinyl]-1H-benzimidazole |
| Steroidal anti-androgen | Cyproterone acetate | 427-51-0 | 9496 | (1R,3aS,3bR,7aR,8aS,8bS,8cS,10aS)-1-Acetyl-5-chloro-8b,10a-dimethyl-7-oxo-1,2,3,3a,3b,7,7a,8,8a,8b,8c,9,10,10a-tetradecahydrocyclopenta[a]cyclopropa[g]phenanthren-1-yl acetate |
| Steroidal anti-asthmatic | Beclomethasone | 4419-39-0 | 19276 | (8S,9R,10S,11S,13S,14S,16S,17R)-9-chloro-11,17-dihydroxy-17-(2-hydroxyacetyl)-10,13,16-trimethyl-6,7,8,11,12,14,15,16-octahydrocyclopenta[a]phenanthren-3-one |
|  | Beclomethasone dipropionate | 08-09-34 | 20396 | [2-[(8S,9R,10S,11S,13S,14S,16S,17R)-9-chloro-11-hydroxy-10,13,16-trimethyl-3-oxo-17-propanoyloxy-6,7,8,11,12,14,15,16-octahydrocyclopenta[a]phenanthren-17-yl]-2-oxoethyl] propanoate |
| Steroidal anti-inflammatory | Budesonide | 51333-22-3 | 36566 | (4aR,4bS,5S,6aS,6bS,9aR,10aS,10bS)-6b-Glycoloyl-5-hydroxy-4a,6a-dimethyl-8-propyl-4a,4b,5,6,6a,6b,9a,10,10a,10b,11,12-dodecahydro-2H-naphtho[2',1':4,5]indeno[1,2-d][1,3]dioxol-2-one |
|  | Clobetasol propionate | 25122-46-7 | 30399 | [(8S,9R,10S,11S,13S,14S,16S,17R)-17-(2-chloroacetyl)-9-fluoro-11-hydroxy-10,13,16-trimethyl-3-oxo-6,7,8,11,12,14,15,16-octahydrocyclopenta[a]phenanthren-17-yl] propanoate |
|  | Clobetasone-17-butyrate | 25122-57-0 | 64481 | [(8S,9R,10S,13S,14S,16S,17R)-17-(2-chloroacetyl)-9-fluoro-10,13,16-trimethyl-3,11-dioxo-7,8,12,14,15,16-hexahydro-6H-cyclopenta[a]phenanthren-17-yl] butanoate |
|  | Dexamethasone | 50-02-2 | 5541 | (8S,9R,10S,11S,13S,14S,16R,17R)-9-fluoro-11,17-dihydroxy-17-(2-hydroxyacetyl)-10,13,16-trimethyl-6,7,8,11,12,14,15,16-octahydrocyclopenta[a]phenanthren-3-one |
|  | Dexamethasone-21-acetate | 1177-87-3 | 206624 | [2-[(8S,9R,10S,11S,13S,14S,16R,17R)-9-fluoro-11,17-dihydroxy-10,13,16-trimethyl-3-oxo-6,7,8,11,12,14,15,16-octahydrocyclopenta[a]phenanthren-17-yl]-2-oxoethyl] acetate |
|  | Flumethasone | 2135-17-3 | 15632 | (6S,8S,9R,10S,11S,13S,14S,16R,17R)-6,9-difluoro-11,17-dihydroxy-17-(2-hydroxyacetyl)-10,13,16-trimethyl-6,7,8,11,12,14,15,16-octahydrocyclopenta[a]phenanthren-3-one |
|  | Fluticasone-17-Propionate | 80474-14-2 | 49297 | [(6S,8S,9R,10S,11S,13S,14S,16R,17R)-6,9-difluoro-17-(fluoromethylsulfanylcarbonyl)-11-hydroxy-10,13,16-trimethyl-3-oxo-6,7,8,11,12,14,15,16-octahydrocyclopenta[a]phenanthren-17-yl] propanoate |
|  | Fusidic acid | 1859-24-0 | 2271900 | (2Z)-2-[(3R,4S,5S,8S,9S,10S,11R,13R,14S,16S)-16-acetyloxy-3,11-dihydroxy-4,8,10,14-tetramethyl-2,3,4,5,6,7,9,11,12,13,15,16-dodecahydro-1H-cyclopenta[a]phenanthren-17-ylidene]-6-methylhept-5-enoic acid    **continued** |
|  | Hydrocortisone | 50-23-7 | 5551 | (8S,9S,10R,11S,13S,14S,17R)-11,17-dihydroxy-17-(2-hydroxyacetyl)-10,13-dimethyl-2,6,7,8,9,11,12,14,15,16-decahydro-1H-cyclopenta[a]phenanthren-3-one |
|  | Hydrocortisone-21-acetate | 50-03-3 | 5542 | [2-[(8S,9S,10R,11S,13S,14S,17R)-11,17-dihydroxy-10,13-dimethyl-3-oxo-2,6,7,8,9,11,12,14,15,16-decahydro-1H-cyclopenta[a]phenanthren-17-yl]-2-oxoethyl] acetate |
|  | Mometasone furoate | 83919-23-7 | 390091 | [(8S,9R,10S,11S,13S,14S,16R,17R)-9-chloro-17-(2-chloroacetyl)-11-hydroxy-10,13,16-trimethyl-3-oxo-6,7,8,11,12,14,15,16-octahydrocyclopenta[a]phenanthren-17-yl] furan-2-carboxylate |
|  | Prednisolone | 50-24-8 | 5552 | (8S,9S,10R,11S,13S,14S,17R)-11,17-dihydroxy-17-(2-hydroxyacetyl)-10,13-dimethyl-7,8,9,11,12,14,15,16-octahydro-6H-cyclopenta[a]phenanthren-3-one |
| Steroidal contraceptive | Desogestrel | 54024-22-5 | 37400 | (8S,9S,10R,13S,14S,17R)-13-ethyl-17-ethynyl-11-methylidene-1,2,3,6,7,8,9,10,12,14,15,16-dodecahydrocyclopenta[a]phenanthren-17-ol |
| Steroidal estrogen | 17-alpha-estradiol | 57-91-0 | 61840 | (8R,9S,13S,14S,17R)-13-methyl-6,7,8,9,11,12,14,15,16,17-decahydrocyclopenta[a]phenanthrene-3,17-diol |
|  | 17-beta-estradiol | 50-28-2 | 5554 | (8R,9S,13S,14S,17S)-13-methyl-6,7,8,9,11,12,14,15,16,17-decahydrocyclopenta[a]phenanthrene-3,17-diol |
| Steroidal hormone | Progesterone | 57-83-0 | 5773 | (8S,9S,10R,13S,14S,17S)-17-acetyl-10,13-dimethyl-1,2,6,7,8,9,11,12,14,15,16,17-dodecahydrocyclopenta[a]phenanthren-3-one |
| Steroidal progestin | Medroxyprogesterone-17-acetate | 71-58-9 | 6043 | [(6S,8R,9S,10R,13S,14S,17R)-17-acetyl-6,10,13-trimethyl-3-oxo-2,6,7,8,9,11,12,14,15,16-decahydro-1H-cyclopenta[a]phenanthren-17-yl] acetate |
|  | Nor-ethisterone (19-Norethindrone) | 68-22-4 | 5994 | (8R,9S,10R,13S,14S,17R)-17-ethynyl-17-hydroxy-13-methyl-1,2,6,7,8,9,10,11,12,14,15,16-dodecahydrocyclopenta[a]phenanthren-3-one |
| Steroidal reductase inhibitor | Finasteride | 98319-26-7 | 51714 | (1S,3aS,3bS,5aR,9aR,9bS,11aS)-N-tert-butyl-9a,11a-dimethyl-7-oxo-1,2,3,3a,3b,4,5,5a,6,9b,10,11-dodecahydroindeno[5,4-f]quinoline-1-carboxamide |
| Vasodilator | Betahistine | 5638-76-6 | 2276 | N-methyl-2-pyridin-2-ylethanamine |
| α1a adrenergic receptor antagonist | Tamsulosin | 106133-20-4 | 114457 | 5-[(2R)-2-[2-(2-ethoxyphenoxy)ethylamino]propyl]-2-methoxybenzenesulfonamide |
|  |  |  |  |  |
| **Key** |  |  |  |  |
| CASRN - Chemical Abstracts Service Registry Number (<https://www.cas.org/content/chemical-substances>) | | | |  |
| Chemspider - Royal Society of Chemistry (<http://www.chemspider.com/)> | | |  |  |
| IUPAC - International Union of Pure and Applied Chemistry (<http://iupac.org/>) | | |  |  |
| PubChem - National Center for Biotechnology Information (<https://pubchem.ncbi.nlm.nih.gov/>) | | | |  |

**Stock and diluted pharmaceutical standard solutions**

Stock solutions (1.0 mg mL^-1^) were prepared by dissolving the standard pharmaceutical compounds in either acetone, acetonitrile, dimethylformamide (DMF), dimethyl sulfoxide (DMSO), ethanol, methanol, sodium hydroxide solution or water depending on the physicochemical properties of the substance [1].

**Acetone:**

17-Alpha-estradiol, 17-Beta-estradiol, Beclomethasone di-propionate, Betamethsone-17-valerate, Bisacodyl, Cinnarizine, Clobetasone-17-butyrate, Cyproterone acetate, Dexamethasone, Felodipine, Fenofibrate, Flumethasone, Hydrocortisone-21-acetate, Hydroxyzine, Ketoprofen, Medroxyprogesterone -17-acetate, Mometasone furoate, Naproxen, Nifedipine, Nitrofurantoin, Norethisterone and Warfarin were dissolved in acetone.

**Ethanol:**

Carvedilol, Diltiazem-*cis*, Dipyridamole, Dosulepin, Finasteride, Hydrocortisone, Losartan, Progesterone, Promethazine, Salbutamol, Scopolamine, Simvastatin, Spironolactone, Terbutaline and Verapamil were dissolved in ethanol.

**Water:**

Cetirizine, Dioctyl sulfosuccinate, Flecainide, Hydroxychloroquine, Penicillin V and Trazadone were dissolved in water.

**Acetonitrile:**

Chlorhexidine and Donepezil were dissolved in acetonitrile.

Nystatin, Doxazosin and Mefenamic acid were dissolved in dimethylformamide, dimethylsulfoxide and 1 M sodium hydroxide respectively.

All remaining pharmaceutical compounds were dissolved in methanol.

Many of the standard compounds were available only as salts e.g. chloride or acetate forms. Standards were made taking into consideration the percentage of salt or water of crystallization. The individual stock standard solutions were stored until use at –18 °C.

A diluted stock standard solution was prepared in methanol at a concentration of 1.0 µg mL^-1^ and was found to be stable for at least one month at 3-5 °C. This solution was diluted 100-fold in mobile phase and used as a working standard solution mix, being prepared when required.

**Creation of the searchable compound database library using Agilent software**

Accurate mass, mass spectra for the protonated adducts [M+H]^+^ in positive ion mode and de-protonated adducts [M-H]^-^ for each pharmaceutical compound were acquired using flow injection in MS mode with a collision energy of 0 eV.

Accurate mass, mass spectra were also acquired for the [M+Na]^+^ and [M+K]^+^ adducts in positive ion mode, or [M-HCOO]^-^ and (M+CH_3_COO]^-^ adducts in negative ion mode, when they were observed in significant abundance.

Accurate mass, fragment ion spectra for the protonated and de-protonated adducts were then acquired using flow injection in MS/MS mode with collision energies of 20 eV and 40 eV.

Fragment ion spectra for other adducts, especially those for sodium and potassium were not always obtained as they tend to be unstable [2,3].

To eliminate mass assignment errors, fragment masses in the acquired spectra were compared with the theoretical fragment formulas and where necessary corrected to their theoretical masses and possible structures.

Theoretical masses for fragment ions and potential structures were generated using ACD Labs Mass Fragmenter software that calculates theoretical fragmentation pathways for compounds under specific ionisation conditions [4].

A searchable compound database library was created by populating it with the accurate mass MS and MS/MS data, acquired above, for the 164 target compounds. MS/MS spectra below 1% of the base peak for each collision energy were removed from the database. In addition, the compound database library was populated with compound information including: the name, formula, accurate mass, structure, database identifiers such as the CAS number and Chemspider ID number and retention times which were obtained from chromatographic analysis of the working standard solution mix of pharmaceuticals.

The mass spectra of the following pharmaceuticals, obtained from the analysis of pure standards, showed significant abundances of adducts of potassium and ammonium that were at least 50% of the base peak (typically the sodiated adduct): bendroflumethiazide, calcipotriol, cefalexin, chloramphenicol, digoxin, docusate, hydrochlorthiazide, hydrocortisone-21-acetate, ketoprofen, nitrofurantoin, orlistat, pravastatin and spironolactone.

However, with the exception of one compound, docusate, the use of adducts of potassium and ammonium when screening for pharmaceuticals in POCIS or Chemcatcher® extracts against an accurate mass database did not result in an increase in the number of compounds identified or in the score obtained over the use of only protonated and sodiated adducts. Use of only adducts of potassium and ammonium in the positive ion accurate mass database search produced low scores, typically well below the set acceptable threshold. In addition, the mass spectra for the above compounds in POCIS and Chemcatcher® extracts did not show the same ratio of sodium to potassium and ammonium as observed in standards. It was decided, therefore, not to include potassium and ammonium adducts in any further investigations.

**Table S2** Chromatographic conditions

Analytical column: Waters, Atlantis T3 Column 2.1 × 150 mm, 3.5 μm particle size

Column temperature: 40 °C

Mobile phase:

A) 2 mM ammonium acetate + 0.01% formic acid in water

B) 2 mM ammonium acetate + 0.01% formic acid in methanol

Gradient programme: Time (min) % B

00.0 5

25.0 100

30.0 100

30.1 5

Stop time: 30.0 min

Post time: 10.0 min

Injection volume: 20 μL

**Table S3** Mass spectrometer conditions

***Positive ion Negative ion***

Nebulizer (psi): 50 45

Sheath gas temperature (°C): 350 300

Sheath gas flow (L/min): 12 11

Capillary voltage (V): 3,000 2,000

Nozzle voltage (V): 1,000 750

Fragmentor voltage (V): 135 110

Skimmer 1 voltage (V) 65 55

Octopole RF Peak 750 750

**MS mode (low energy channel, 0 eV)**

Data acquisition scan rate (Hz) 1 1

Mass range (Da) 50-1,100 50-1,100

**All Ions MS/MS mode (high energy channel, 20 & 40 eV)**

Data acquisition scan rate (Hz) 3 3

Mass range (Da) 50-1,100 50-1,100

**Compound extraction of targeted pharmaceuticals**

A compound database library of 164 pharmaceuticals was developed and used in conjunction with Agilent Mass Hunter Profinder software to identify pharmaceuticals in the Chemcatcher^®^ and POCIS extracts. A process within the software called ‘Batch Targeted Feature Extraction’ was used to extract and identify compounds from the within the compound database library based on their chemical formulae. A mass error setting of 10 ppm for the molecular adduct ion and fragment ions was used with additional confirmation obtained from comparing the isotope abundance pattern and isotope spacing mass accuracy. The parameters used are given in Table S4.

**Table S4** Parameters used for the ‘Batch Targeted Feature Extraction’ of compounds in extracts from POCIS and Chemcatcher® samplers

| **Source of formulas to confirm** |  |
| --- | --- |
| Database: | Pharmaceuticals |
| Values to match: | Mass and retention time |
|  |  |
| **Charge carriers (adducts)** |  |
| Positive Ions | Negative Ions |
| H^+^ (protonated) | H^-^ (deprotonated) |
| Na^+^ (sodiated) | HCOO^-^ (formate) |
|  | CH_3_COO^-^ (acetate) |
|  |  |
| **Isotope grouping** |  |
| Peak spacing tolerance: | 0.0025 m/z, plus 7 ppm |
| Isotope model: | Common organic molecules |
| Charge state maximum: | 1 |
|  |  |
| **Matching tolerances and scoring** |  |
| Mass match tolerance: | +/- 10 ppm |
| Retention time tolerance: | +/- 1.0 min |
|  |  |
| **Contribution to overall score** |  |
| Mass score: | 100 |
| Isotope abundance score: | 60 |
| Isotope spacing score: | 50 |
| Retention time score: | 100 |
|  |  |
| **Matching criteria** |  |
| Do not match if score is less than: | 60 |
| **Spectra to include** |  |
| Average scans: | At 25% of peak height |
| Peak spectrum background: | Average of spectra at peak start and end |
|  |  |
| **Post processing filters** |  |
| Minimum height: | 1000 counts |
| Minimum filter matches: | Compound must be present across all data files |

**Table S5** Analyses of variance of the log_10_ transformed integrated peak areas for 2982 unknown compounds found at all sites (including duplicate deployment at WWTP 2 site) using the Chemcatcher® and POCIS.

Variate: log_10_ (integrated peak areas) Chemcatcher® all sites

| **Source of variation** | **d.f.** | **s.s.** | **m.s.** | **v.r.** | **F pr.** |
| --- | --- | --- | --- | --- | --- |
| **Site** | 3 | 4.215E+00 | 1.405E+00 | 274.55 | <.001 |
| **Compound** | 732 | 2.022E+03 | 2.762E+00 | 539.83 | <.001 |
| **Site.Compound** | 2196 | 1.040E+02 | 4.735E-02 | 9.25 | <.001 |
| **Residual** | 5864 | 3.001E+01 | 5.117E-03 |  |  |
| **Total** | 8795 | 2.160E+03 |  |  |  |

Variate: log_10_ (integrated peak areas) POCIS all sites

| **Source of variation** | **d.f.** | **s.s.** | **m.s.** | **v.r.** | **F pr.** |
| --- | --- | --- | --- | --- | --- |
| **Site** | 3 | 9.493E+00 | 3.164E+00 | 321.44 | <.001 |
| **Compound** | 732 | 1.910E+03 | 2.609E+00 | 265.07 | <.001 |
| **Site.Compound** | 2196 | 1.199E+02 | 5.462E-02 | 5.55 | <.001 |
| **Residual** | 5864 | 5.772E+01 | 9.844E-03 |  |  |
| **Total** | 8795 | 2.097E+03 |  |  |  |

The residual variance (MS) gives a measure of the variation between replicates after the differences between compounds, sites, and an interaction between compounds and site had been removed. The interaction term represents the differences in pattern of concentrations of different compounds between the sites.

**Table S6** Statistical output from the log_10_ transformed orthogonal regression analysis for the 68 pharmaceutical compounds found at all sites (including duplicate deployment at WWTP 2 site) using the Chemcatcher® and POCIS.

**Error Variance Ratio:** log_10_POCIS/log_10_Chemcatcher: 1.92

**Regression Equation:** log_10_POCIS = 0.264 + 1.022 log_10_Chemcatcher

**Coefficients:**

| **Predictor** | **Coefficient** | **SE Coefficient** | **Z** | **P** | **Approx 95% CI** |
| --- | --- | --- | --- | --- | --- |
| **Constant** | 0.26449 | 0.0448905 | 5.8920 | 0.000 | (0.17651, 0.35248) |
| **Log_10_Chemcatcher** | 1.02166 | 0.0083305 | 122.6406 | 0.000 | (1.00533, 1.03798) |

**Error Variances:**

| **Variable** | **Variance** |
| --- | --- |
| **Log_10_POCIS** | 0.0196024 |
| **Log_10_Chemcatcher** | 0.0102096 |

**Table S7** Three way analysis of variance of the log_10_ integrated peak areas for 68 pharmaceutical compounds accumulated in the Chemcatcher® and POCIS samplers in the four deployments. The treatments were pharmaceutical, site and sampler. Every interaction term was included.

| **Source of variation** | **d.f.** | **s.s.** | **m.s.** | **v.r.** | **F pr.** |
| --- | --- | --- | --- | --- | --- |
| **Sampler** | 1 | 58.950578 | 58.950578 | 11980.34 | <.001 |
| **Compound** | 67 | 868.451648 | 12.961965 | 2634.22 | <.001 |
| **Site** | 3 | 4.274648 | 1.424883 | 289.57 | <.001 |
| **Sampler.compound** | 67 | 6.293854 | 0.093938 | 19.09 | <.001 |
| **Sampler.site** | 3 | 0.926021 | 0.308674 | 62.73 | <.001 |
| **Compound.site** | 201 | 39.911287 | 0.198564 | 40.35 | <.001 |
| **Sampler.compound.site** | 201 | 2.215267 | 0.011021 | 2.24 | <.001 |
| **Residual** | 1088 | 5.353624 | 0.004921 |  |  |
| **Total** | 1631 | 986.376926 |  |  |  |

* d.f. (degrees of freedom), s.s. (sums of squares), m.s. (mean square), v.r. (variance ratio) and F pr. (probability associated with the F value)

**Table S8** Multiple comparison between site means using a Bonferroni test with means in ascending order. The means with the same letter were not significantly different at the 5% level of probability.

| **Site** | **Log_10_ mean** | **Untransformed mean** | **Significant differences** |
| --- | --- | --- | --- |
| **B2** | 5.489 | 308,000 | A |
| **B1** | 5.504 | 319,000 | B |
| **C** | 5.505 | 320,000 | B |
| **A** | 5.617 | 414,000 | C |

**Table S9** Analysis of variance of the differences between log_10_ integrated peak areas for 68 pharmaceutical compounds accumulated in the Chemcatcher® samplers in two parallel deployments at WWTP B site. The factors used were deployment and compound, and an interaction term was calculated.

Variate: log_10_ uptake Chemcatcher®

| **Source of variation** | **d.f.** | **s.s.** | **m.s.** | **v.r.** | **F pr.** |
| --- | --- | --- | --- | --- | --- |
| **Cage_no** | 1 | 0.05042 | 0.05042 | 1.24 | 0.267 |
| **Compound** | 66 | 223.94974 | 3.39318 | 83.28 | <.001 |
| **Cage_no.Compound** | 66 | 0.70252 | 0.01064 | 0.26 | 1.000 |
| **Residual** | 274 | 11.16395 | 0.04074 |  |  |
| **Total** | 407 | 235.86662 |  |  |  |

* d.f. (degrees of freedom), s.s. (sums of squares), m.s. (mean square), v.r. (variance ratio) and F pr. (probability associated with the F value)

**Table S10** Analysis of variance of the differences between log_10_ integrated peak areas for 68 pharmaceutical compounds accumulated in the POCIS samplers in two parallel deployments at WWTP B site. The factors used were deployment and compound, and an interaction term was calculated.

Variate: log_10_ uptake POCIS

| **Source of variation** | **d.f.** | **s.s.** | **m.s.** | **v.r.** | **F pr.** |
| --- | --- | --- | --- | --- | --- |
| **Cage_no** | 1 | 0.28396 | 0.28396 | 6.29 | 0.013 |
| **Compound** | 66 | 235.52185 | 3.56851 | 79.02 | <.001 |
| **Cage_no.Compound** | 66 | 1.05725 | 0.01602 | 0.35 | 1.000 |
| **Residual** | 274 | 12.37298 | 0.04516 |  |  |
| **Total** | 407 | 249.23605 |  |  |  |

* d.f. (degrees of freedom), s.s. (sums of squares), m.s. (mean square), v.r. (variance ratio) and F pr. (probability associated with the F value)


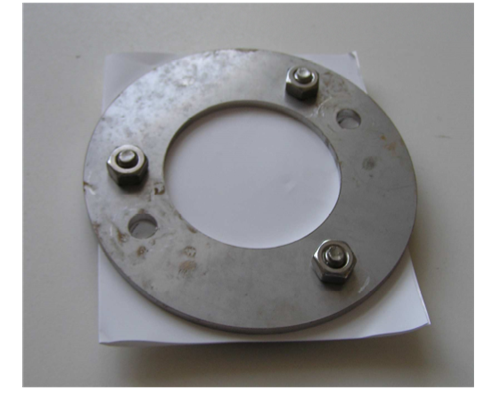


**Fig. S1** Photograph of the POCIS device being assembled


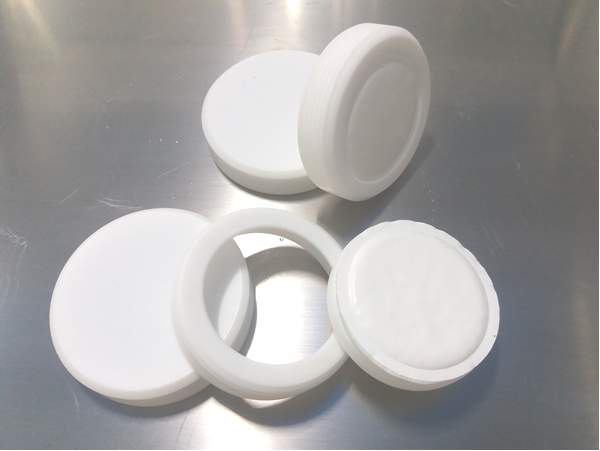


**Fig. S2** Photograph of the three component Chemcatcher® body (top assembled and bottom dissembled) with HLB-L receiving phase disk and PES membrane

**
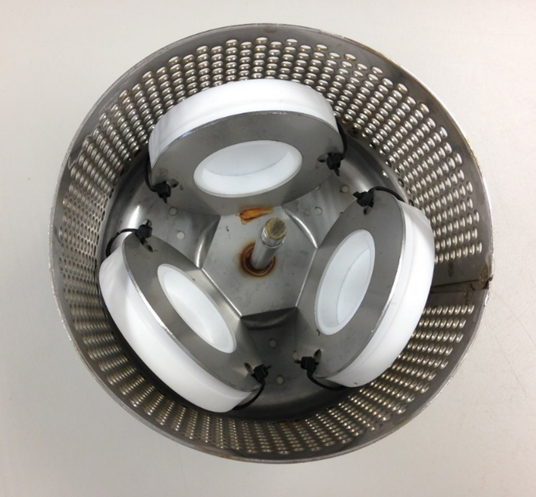
**

**Fig. S3** Photograph of triplicate Chemcatcher® samplers held on holder in deployment canister


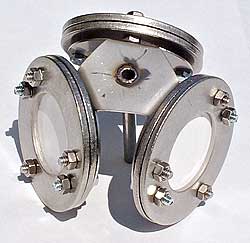


**Fig. S4** Photograph of triplicate POCIS held on holder ready to deploy in deployment canister

**
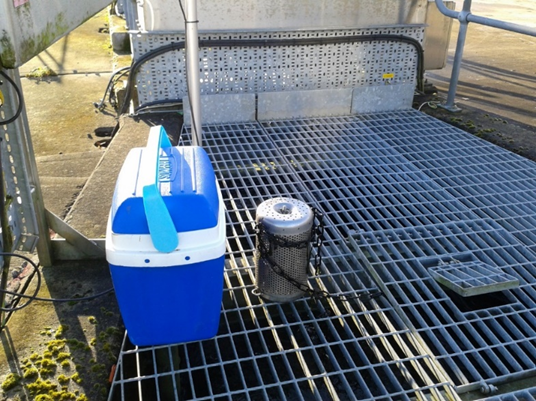
**

**Fig. S5** Photograph of deployment of passive samplers in protective canister at the final effluent channel at WWTP site B

**(a)**


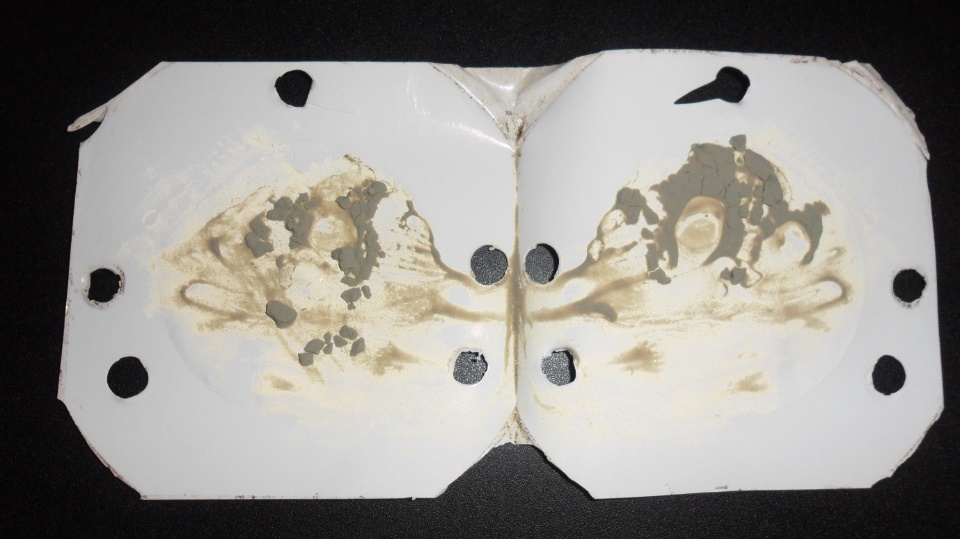


**
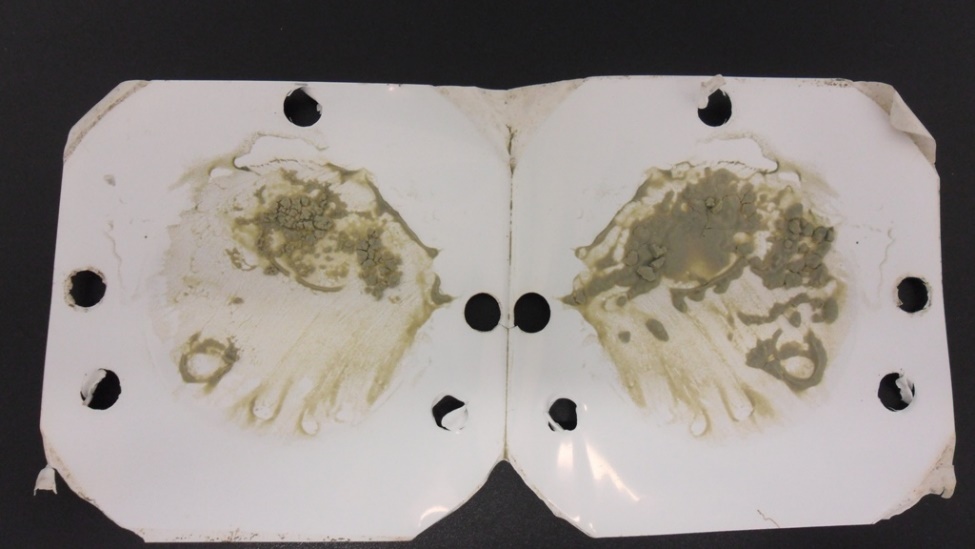
**

**(b)**

**Fig. S6** Photographs of the distribution of HLB sorbent within a POCIS after disassembly. Each sampler deployed at (a) WWTP site B (deployment 1) and (b) WWTP site B (deployment 2). There was evidence that the HLB sorbent sagged towards the base of the sampler during deployment in the vertical plane.


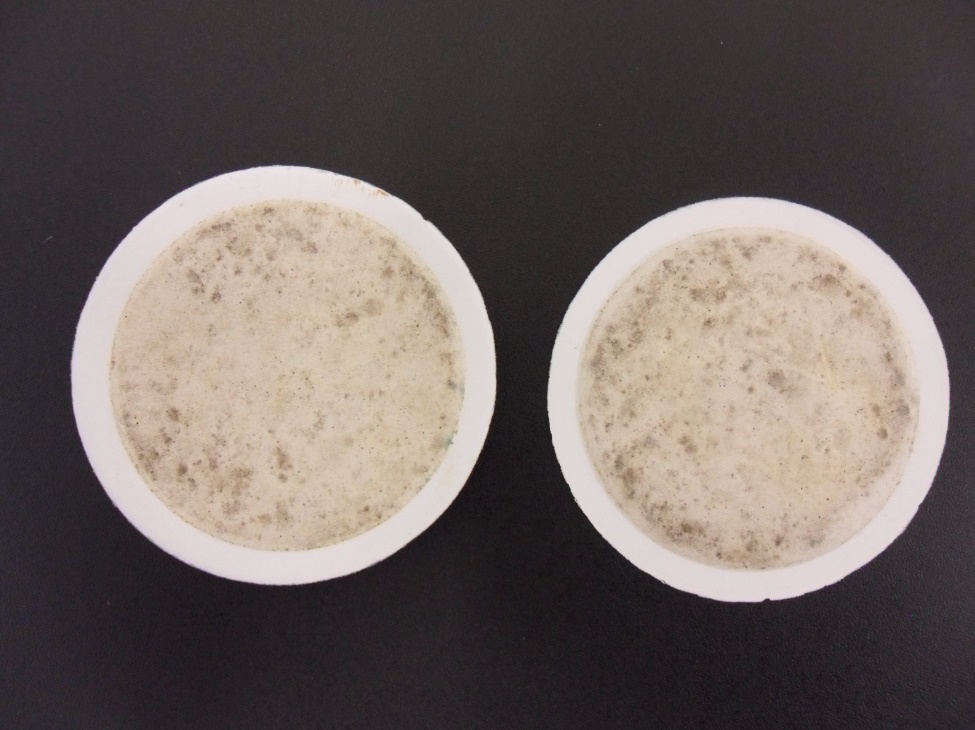
**(a)**

**
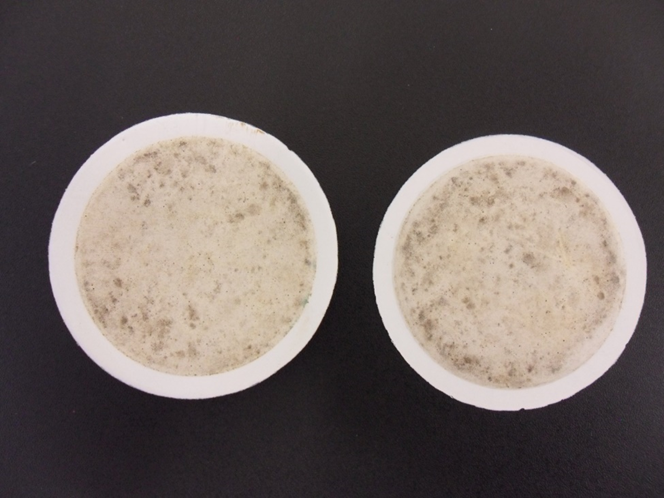
(b)**

**Fig. S7** Photographs of HLB-L disks from retrieved Chemcatcher® sampler. Each sampler deployed at (a) WWTP site B (deployment 1) and (b) WWTP site B (deployment 2).


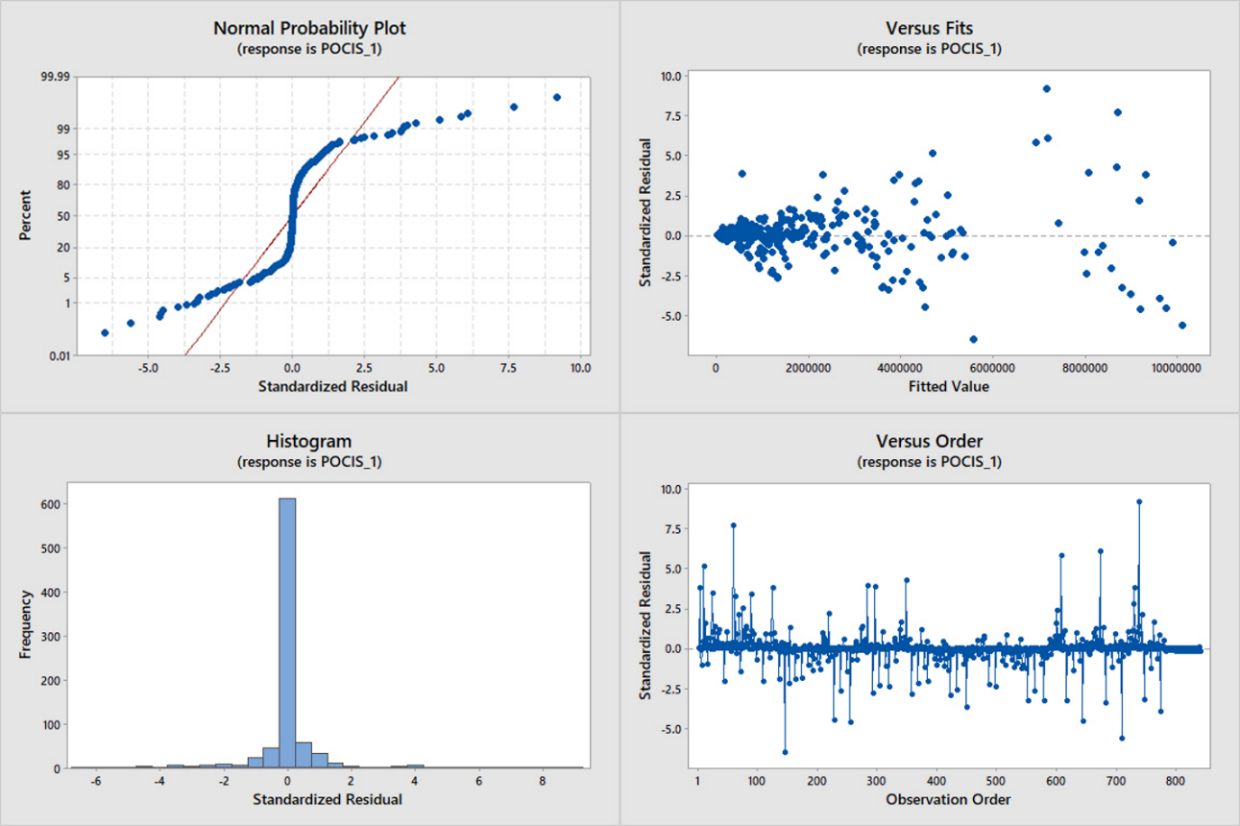


**Fig. S8** Plots of standardised residuals obtained from the orthogonal regression analysis of non-transformed data for the 68 pharmaceutical compounds found at all sites (including the duplicate deployment at site WWTP 2) using the Chemcatcher® and POCIS.


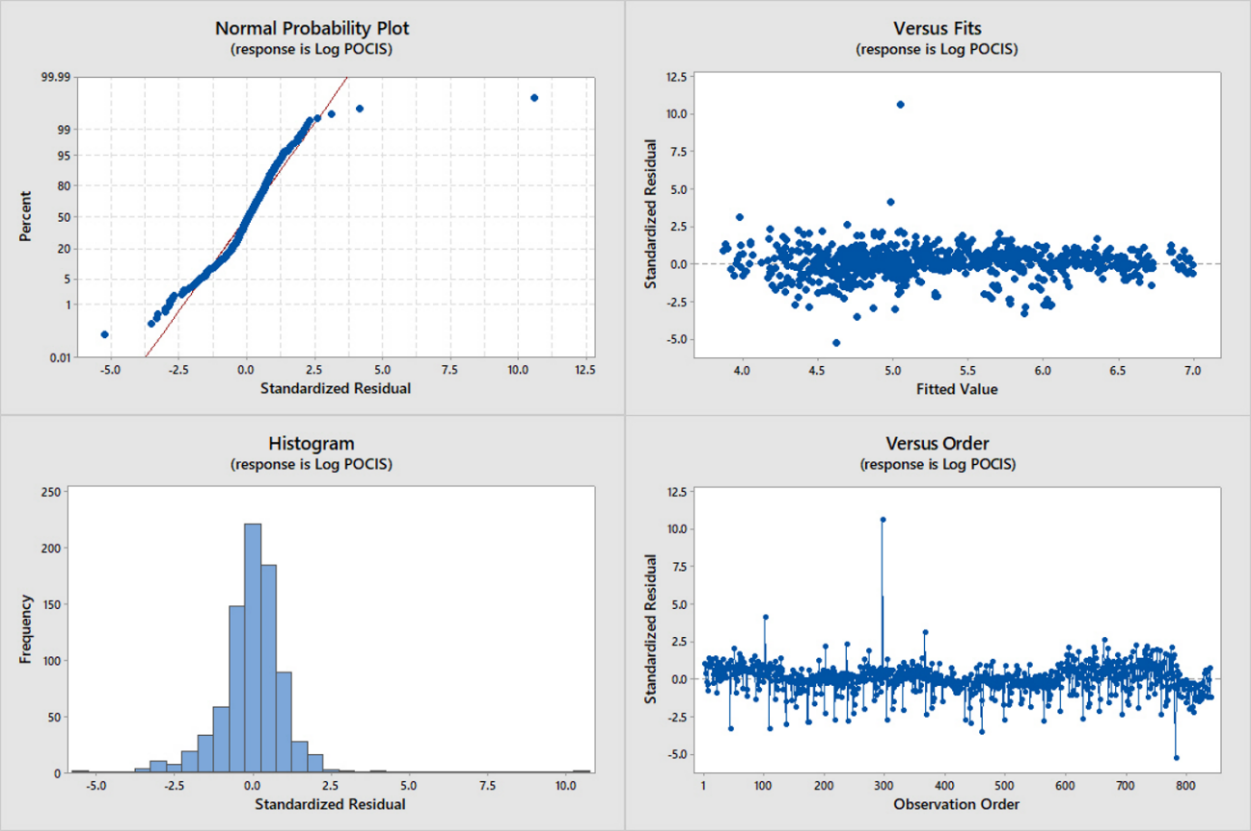


**Fig. S9** Plots of standardised residuals obtained from the orthogonal regression analysis of log_10_ transformed data for the 68 pharmaceutical compounds found at all sites (including the duplicate deployment at site WWTP 2) using the Chemcatcher® and POCIS.


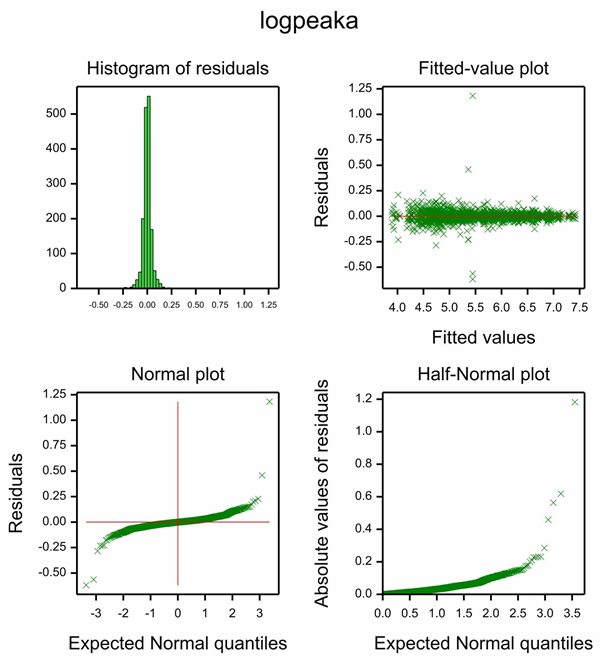


**Fig. S10** Plots of standardised residuals obtained from the three-way analysis of variance of the orthogonal regression analysis of log_10_ transformed data for the 68 pharmaceutical compounds found at all sites (including the duplicate deployment at site WWTP 2) using the Chemcatcher® and POCIS.

**References:**

[1] A. Jouyban, Handbook of solubility data for pharmaceuticals, CRC Press, 2009.

[2] M. Schumacher, G. Castle, A. Gravell, G.A. Mills, G.R. Fones, An improved method for measuring metaldehyde in surface water using liquid chromatography tandem mass spectrometry, MethodsX, 3 (2016) 188-194, doi:10.1016/j.mex.2016.03.004.

[3] S.M. Gao, Z.P. Zhang, H.T. Karnes, Sensitivity enhancement in liquid chromatography/atmospheric pressure ionization mass spectrometry using derivatization and mobile phase additives, J. Chromatogr. B. 825 (2005) 98-110, doi:10.1016/j.jchromb.2005.04.021.

[4] S. Wolf, S. Schmidt, M. Mueller-Hannemann, S. Neumann, In silico fragmentation for computer assisted identification of metabolite mass spectra, BMC Bioinformatics, 11 (2010), doi:10.1186/1471-2105-11-148.
